# Genome maintenance meets mechanobiology

**DOI:** 10.1007/s00412-023-00807-5

**Published:** 2023-08-15

**Authors:** Vincent Spegg, Matthias Altmeyer

**Affiliations:** Department of Molecular Mechanisms of Disease, University of Zurich, Zurich, Switzerland

**Keywords:** Genome stability, Replication stress, DNA repair, Biomolecular condensates, Telomere maintenance, Mechanobiology

## Abstract

Genome stability is key for healthy cells in healthy organisms, and deregulated maintenance of genome integrity is a hallmark of aging and of age-associated diseases including cancer and neurodegeneration. To maintain a stable genome, genome surveillance and repair pathways are closely intertwined with cell cycle regulation and with DNA transactions that occur during transcription and DNA replication. Coordination of these processes across different time and length scales involves dynamic changes of chromatin topology, clustering of fragile genomic regions and repair factors into nuclear repair centers, mobilization of the nuclear cytoskeleton, and activation of cell cycle checkpoints. Here, we provide a general overview of cell cycle regulation and of the processes involved in genome duplication in human cells, followed by an introduction to replication stress and to the cellular responses elicited by perturbed DNA synthesis. We discuss fragile genomic regions that experience high levels of replication stress, with a particular focus on telomere fragility caused by replication stress at the ends of linear chromosomes. Using alternative lengthening of telomeres (ALT) in cancer cells and ALT-associated PML bodies (APBs) as examples of replication stress-associated clustered DNA damage, we discuss compartmentalization of DNA repair reactions and the role of protein properties implicated in phase separation. Finally, we highlight emerging connections between DNA repair and mechanobiology and discuss how biomolecular condensates, components of the nuclear cytoskeleton, and interfaces between membrane-bound organelles and membraneless macromolecular condensates may cooperate to coordinate genome maintenance in space and time.

## Introduction to cell cycle regulation

**Fig. 1:**
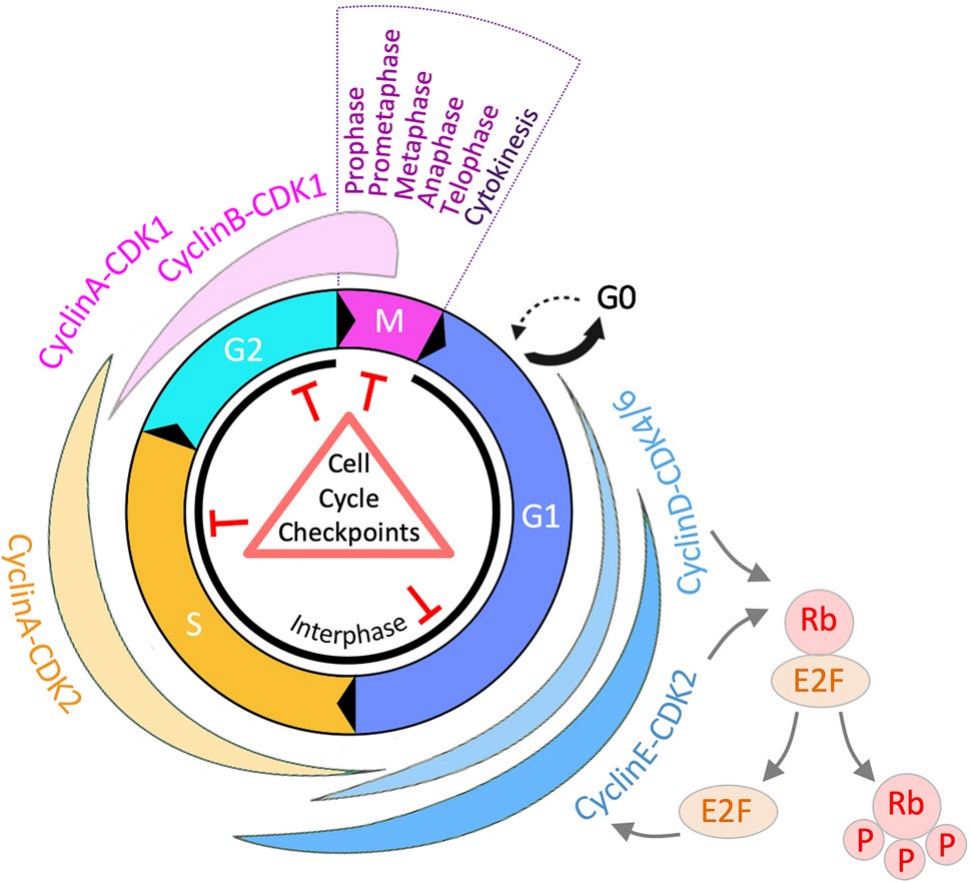
Overview of the eukaryotic cell cycle. Illustration of the different cell cycle phases during interphase (G1, S, G2) and mitosis (M; subdivided into prophase, prometaphase, metaphase, anaphase, telophase, and cytokinesis). The associated Cyclin-CDK activities that drive cell cycle progression and the main cell cycle checkpoints are indicated. Cell cycle exit into quiescence (G0) occurs under unfavorable growth condition. Upon mitogen stimulation, phosphorylation of the negative regulator Rb by CyclinD-CDK4/6 in G1 activates a positive feedback loop centered around the transcription factor E2F and E2F-dependent CyclinE-CDK2 activation. G1, Gap 1; S, S-phase; G2, Gap 2; G0, Gap 0; M, mitosis; Rb, retinoblastoma protein; P, phosphate group; E2F, E2 promoter binding factor; CDK, cyclin-dependent kinase

The eukaryotic cell cycle comprises a series of tightly controlled events that culminate in cell division and in the generation of two new daughter cells (Figure 1). It can be divided into the two main stages mitosis (M-phase) and interphase. While mitosis refers to the process of chromosome segregation followed by cell division, interphase separates two M-phases and provides the time needed for genome duplication and to prepare for cell division. Interphase can be subdivided further into three consecutive cell cycle phases, which are called Gap 1 (G1), Synthesis (S), and Gap 2 (G2). During G1, cells grow and produce proteins and organelles for cellular metabolism and to prepare for later cell cycle phases. G1 length varies depending on growth conditions and intracellular and extracellular cues. In unfavorable conditions, cells may exit the cell cycle in G1 and enter a non-proliferative state known as quiescence or Gap 0 (G0). Following S-phase commitment in favorable growth conditions, cells duplicate their genome by DNA synthesis. After two identical copies of the genetic material have been generated through semi-conservative DNA replication, the ensuing G2-phase serves as an additional gap phase for protein synthesis and cell growth in preparation for mitosis. Finally, in M-phase, the two sets of chromosomes are first condensed and aligned at the equatorial metaphase plate and then segregated as cells divide into two newly emerging daughter cells. M-phase can be subdivided morphologically and functionally into prophase, prometaphase, metaphase, anaphase, telophase, and cytokinesis (Figure 1).

The central regulators of the cell cycle are cyclin-dependent kinases (CDKs). CDKs are serin/threonine kinases whose activation state determines cell cycle entry, progression, and completion (Barnum & O’Connell 2014; Basu et al. 2022; Matthews et al. 2022). Small proteins called cyclins are needed as regulatory subunits of CDKs to stimulate their kinase activity. Cyclins accumulate during different stages of the cell cycle in a manner that is controlled by their cell cycle-dependent expression and targeted proteasomal degradation. A regulatory feed-forward loop between cyclin-dependent CDK activity and CDK-driven cell cycle progression ensures that cell cycle transitions occur in a unidirectional and sequential manner (Novak et al. 2007; Pennycook & Barr 2020).

After cell division, accumulation of CyclinD-CDK4/6 upon exposure to mitogenic growth factors promotes cell cycle commitment and prevents quiescence. CDK4/6 activity fuels E2F-dependent gene expression, which in turn leads to the accumulation of CyclinE and promotes progression through G1 and towards the G1/S transition (Rubin et al. 2020). CyclinE-CDK2 activity further stimulates E2F-dependent transcription by phosphorylating and inactivating the transcription inhibitor retinoblastoma protein (Rb), thereby amplifying the E2F transcriptional signal and generating a positive feedback loop (Figure 1). Inactivation of the APC/C ubiquitin ligase complex at the G1/S transition allows CyclinA-CDK2 activity to rise and initiate DNA replication in S-phase. Following DNA replication, accumulation of CyclinA/B-CDK1 in G2 eventually drives mitotic entry and allows APC/C reactivation, which is required for targeted degradation of S/G2-phase cyclins and mitotic exit to complete the cell cycle (Matthews et al. 2022; Pennycook & Barr 2020).

Several cell cycle checkpoints have evolved to ensure error-free progression through the cell cycle (Elledge 1996; Hartwell & Weinert 1989; Kastan & Bartek 2004). These checkpoints serve to monitor cell cycle progression and to actively slow down or halt the cell cycle upon encountering problems (Figure 1). Cell cycle checkpoint activation can also trigger cell death upon persistent perturbation and thereby guard against cellular transformation. DNA replication stress during S-phase progression and replication stress-induced DNA damage are important sources for cell cycle checkpoint activation (Gaillard et al. 2015; Nyberg et al. 2002). In the following paragraphs we summarize the processes that are central for DNA replication and for the cellular response to replication stress.

## DNA replication in eukaryotes

**Fig. 2:**
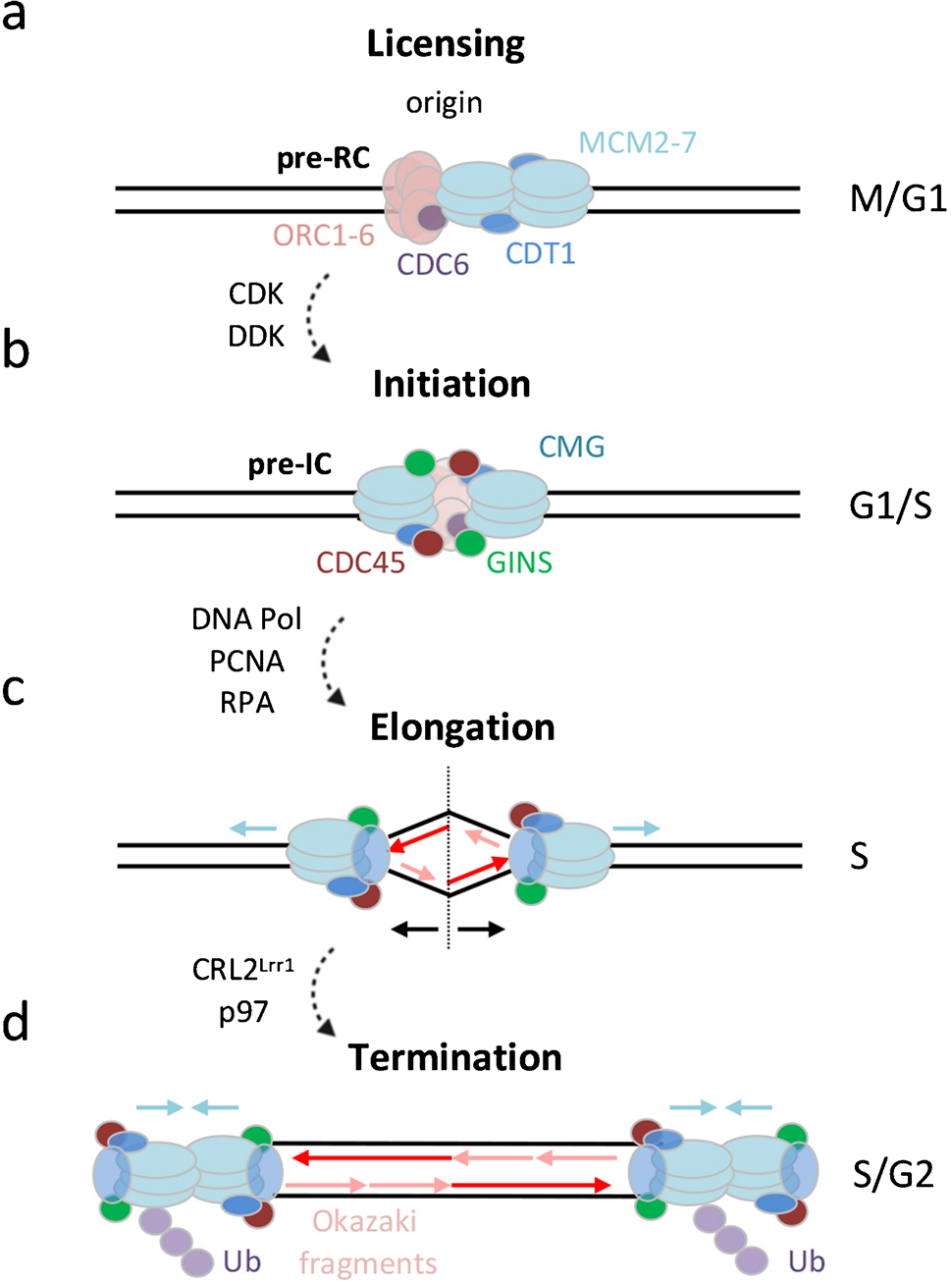
Overview of DNA replication. Indicated are the four phases of DNA replication and their timing during the cell cycle. (a) Origin licensing in late M and early G1. Formation of the pre-replication complex (pre-RC) by the assembly of ORC1-6, CDC6, CDT1, and MCM2-7 at the origin of replication. (b) Replication initiation at the G1/S transition. CDK- and DDK-mediated phosphorylation of the MCM complex leads to the recruitment of CDC45 and the GINS complex, which together with the MCM proteins form the CDC45-MCM-GINS (CMG) helicase, thereby setting up the pre-initiation complex (pre-IC). (c) Elongation during S-phase progression. Recruitment of additional replication proteins, including PCNA, RPA, and DNA polymerases (DNA Pol), leads to the transition from the pre-IC into two active replisomes moving in opposite directions. (d) Termination of replication in S/G2 when two replisomes converge, followed by CRL2^Lrr1^-dependent ubiquitylation of MCM7 and p97-mediated extraction from the DNA. M, mitosis: G1, Gap 1; S, S-phase; G2, Gap 2; CDK, cyclin-dependent kinase; DDK, DBF4-dependent kinase; Ub, ubiquitin

DNA replication in S-phase is tightly regulated, ensuring that the genome is fully copied once and only once in every cell cycle. The process of semi-conservative DNA replication, after which each of the two generated DNA copies contains parental and newly synthesized strands, can be divided into four distinct phases: licensing, initiation, elongation, and termination (Figure 2).

### Licensing

DNA replication begins at defined sites in the genome, called origins of replication. In human cells, DNA replication is initiated from multiple origins of replication and an estimated 30,000–50,000 origins are active in each cell cycle of a replicating cell (Leonard & Mechali 2013). These origins are not all initiated simultaneously at the beginning of S-phase, instead they are activated sequentially during S-phase progression (Boos & Ferreira 2019; Vouzas & Gilbert 2021). A defined consensus sequence that determines replication origins and their temporal activation program in mammals has remained elusive, and it seems that replication initiation is influenced by DNA topology, chromatin organization and transcriptional activity (Emerson et al. 2022; Ganier et al. 2019; Giles et al. 2022; Limas & Cook 2019; Marchal et al. 2019; Prioleau & MacAlpine 2016).

Origin licensing (Figure 2a) occurs before S-phase in late M and early G1 phase of the cell cycle, when the origin recognition complex (ORC), a hexameric protein complex consisting of the subunits ORC1-6, binds to replication origins (Masai et al. 2010). ORC binding leads to the recruitment of the licensing factors CDC6 and CDT1, which facilitates the recruitment of inactive hexameric MCM protein complexes consisting of the proteins MCM2-7 (Nishitani & Lygerou 2002). The origin-associated multiprotein complex formed by ORC, CDC6, CDT1, and MCM2-7 is called pre-replication complex (pre-RC). Two MCM complexes are loaded onto the chromatin head-to-head and form the core of the replicative helicase (Bleichert 2019; Deegan & Diffley 2016; Evrin et al. 2009; Miller et al. 2019; Remus et al. 2009).

Cells load an excess number of pre-RCs and only a subset of licensed origins is used for replication initiation (Masai et al. 2010). Origins have therefore been classified into constitutive, flexible, and dormant origins (Blow et al. 2011; Fragkos et al. 2015). The excess of licensed origins can serve as a backup to complete DNA replication under conditions of replication stress (Blow et al. 2011; Courtot et al. 2018; Fragkos et al. 2015).

The restricted time window for origin licensing in late M and early G1 prevents re-licensing during S-phase. Re-licensing in S-phase could cause re-replication of already copied DNA, leading to amplification of DNA sequences and increased genome instability (Blow & Gillespie 2008; Fragkos et al. 2015; Limas & Cook 2019; Neelsen et al. 2013). The negative regulator of origin licensing Geminin, which is destabilized in G1/M by the APC/C ubiquitin ligase complex, accumulates after APC/C inactivation at the G1/S transition and prevents re-licensing (Machida et al. 2005; Petropoulos et al. 2019). Geminin interacts with the licensing factor CDT1 and blocks loading of the MCM complex, thereby inhibiting the formation of new pre-RCs in S-phase (Lee et al. 2004; Wohlschlegel et al. 2000; Yanagi et al. 2002). A second way of controlling pre-RC formation is mediated by the ubiquitin ligases SCF-Skp2 and DDB1-Cul4, which ubiquitinate CDT1 and ORC1 in S-phase, leading to their proteasomal degradation (Li et al. 2003; Méndez et al. 2002; Nishitani et al. 2006). Finally, in G2 and early M-phase, high CDK activity inactivates pre-RC components, ensuring that licensing of new origins occurs only after chromosome segregation (Machida et al. 2005; Petropoulos et al. 2019).

### Initiation

Licensed origins with pre-RCs are converted to pre-initiation complexes (pre-ICs) upon S-phase entry. This step is regulated by the recruitment of CyclinE-CDK2, DDK (DBF4/CDC7), and CyclinB-CDK1 (Suski et al. 2022), which phosphorylate the MCM complex (Figure 2b). MCM phosphorylation leads to the recruitment of CDC45 and the GINS protein complex, thereby forming the CDC45-MCM-GINS (CMG) helicase (Lewis et al. 2022). CMG complex formation is a prerequisite to activate the MCM helicase activity (Ilves et al. 2010; Zou & Stillman 2000). The protein TopBP1 and its interaction partner Treslin are also part of the pre-IC and bind to the MCM complex in a CDK phosphorylation-dependent manner. Recruitment of TopBP1-Treslin to the MCM complex is required for activation of the CMG helicase (Kumagai et al. 2010, 2011). As a result, two inactive MCM complexes get remodeled into two active CMG complexes, which start to unwind the parental DNA in a bidirectional manner, thereby allowing the recruitment of additional replication proteins, including replication factor c (RFC), proliferating cell nuclear antigen (PCNA), replication protein a (RPA), and DNA polymerases (Limas & Cook 2019; Parker et al. 2017). Together they mediate the transition from a pre-IC into two active replisomes that move in opposite directions and generate the replication bubble (Douglas et al. 2018; Fragkos et al. 2015).

### Elongation

Once DNA replication has been initiated by origin firing, replisomes move away from the replication origin to copy the parental DNA (Figure 2c). Replicative DNA polymerases are incapable of initiating DNA synthesis de novo, but instead need a start site, or primer, to begin DNA synthesis. This primer is synthesized by DNA Polα-primase, which generates a short stretch of ribonucleic acid (RNA) (Arezi & Kuchta 2000). The primer is recognized by RFC, which loads the replication sliding clamp PCNA and displaces DNA Polα-primase with replicative DNA Polδ/ε (Moldovan et al. 2007; O'Donnell et al. 2013). As new deoxyribonucleotides can only be added at the 5’-phosphate ends of nascent DNA strands, one of the two daughter strands is synthesized in a continuous manner in the same direction as the moving replication fork (leading strand synthesis), while the opposite daughter strand must be synthesized away from the replication fork and hence in a discontinuous manner (lagging strand synthesis). For leading strand synthesis DNA Polε needs only one primer formed at the origin, whereas for lagging strand synthesis the process of primer synthesis followed by extension must be repeated periodically. The newly synthesized fragments of the lagging strand are termed Okazaki fragments (Okazaki et al. 1968). Through leading and lagging strand synthesis in conjunction with Okazaki fragment ligation, both parental DNA strands are copied, and continuous stretches of newly synthesized DNA are formed within each replication unit.

### Termination

DNA replication is terminated when converging replication forks coming from two replication units encounter each other (Figure 2d). When converging forks meet, the replisomes disassemble leaving a ssDNA gap between the 3’-end of the leading strand and the downstream Okazaki fragment of the opposing fork. The remaining gap is filled by extension of the leading strand resulting in a continuous DNA molecule (Dewar & Walter 2017). Unloading of the CMG helicase from termination sites was recently shown to require the ubiquitin-selective segregase p97 after polyubiquitylation of MCM7 by CRL2^Lrr1^ (Dewar & Walter 2017; Fan et al. 2021). A backup mechanism seems to exist in mitosis to trigger global replisome disassembly through MCM7 polyubiquitylation by the ubiquitin E3 ligase TRAIP, followed by p97-mediated extraction from the chromatin (Deng et al. 2019; Priego Moreno et al. 2019; Sonneville et al. 2019; Villa et al. 2021). Failure to terminate replication, e.g., due to obstacles that impair replication fork speed, can undermine the faithful propagation of the genetic information to the next cell generation and is associated with DNA replication stress.

## Replication stress

**Fig. 3:**
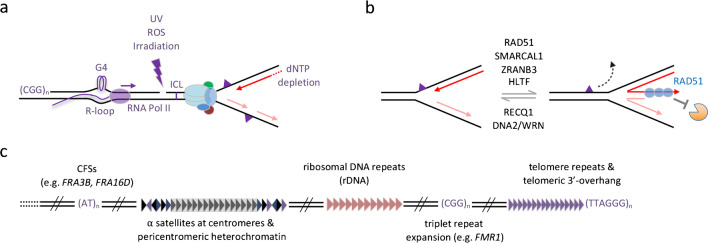
Replication stress and fragile genomic regions. (a) Sources of replication stress that can lead to replication fork stalling and fork collapse. Indicated are exogenous sources of replication stress (e.g., UV light, irradiation) and endogenous sources of replication stress (e.g., repetitive sequences, secondary structures such as G4-quadruplexes and R-loops, reactive oxygen species (ROS), interstrand crosslinks (ICLs), dNTP depletion). (b) Replication fork reversal as a protective mechanism against replication stress. Indicated is the dynamic process of fork reversal and re-reversal for fork restart, as well as main regulators of fork reversal and of reversed fork protection. (c) Fragile genomic regions that are prone to experiencing replication stress. Indicated are common fragile sites (CFSs), centromeric and pericentromeric regions, ribosomal DNA, rare fragile sites of triplet repeat expansion, and telomere repeats

During the process of DNA replication, the replication machinery is confronted with a variety of obstacles that can interfere with DNA synthesis and jeopardize timely completion of genome duplication (Mazouzi et al. 2014; Saxena & Zou 2022). Conditions that lead to replication fork slowing or stalling and perturb DNA synthesis are generally referred to as replication stress (Gaillard et al. 2015; Zeman & Cimprich 2014). Replication stress can be caused by exogenous sources such as DNA-modifying chemicals and alterations of the DNA structure, e.g., through ionizing radiation (IR) or ultraviolet (UV) light (Figure 3a). Endogenous sources of replication stress include depleted deoxyribonucleotide pools, ribonucleotide incorporation into DNA, DNA lesions caused by metabolic byproducts such as reactive oxygen species (ROS), interstrand crosslinks (ICLs), DNA secondary structures such as hairpins and G4-quadruplexes, repetitive DNA sequences, transcription-replication conflicts, and RNA-DNA hybrids (Brickner et al. 2022; Garcia-Muse & Aguilera 2016; Petermann et al. 2022; Saxena & Zou 2022; Zeman & Cimprich 2014). Replication stress has emerged as major cause of genome instability and is a hallmark of most cancers (Macheret & Halazonetis 2015). Considering the elevated levels of replication stress in cancer cells, enzymes involved in the response to replication stress are promising targets for cancer therapy (Cybulla & Vindigni 2023; da Costa et al. 2023; Dobbelstein & Sorensen 2015).

### Replication stress response

Obstacles that lead to stalling of DNA polymerases on the lagging strand are generally well tolerated. Due to the discontinuous nature of Okazaki fragment synthesis, such lesions can be efficiently bypassed, leaving behind short ssDNA gaps, which are repaired post-replicatively (Berti & Vindigni 2016; Marians 2018; Yeeles et al. 2013). On the other hand, stalling of DNA polymerases during leading strand synthesis can cause helicase-polymerase uncoupling and challenge fork stability (Taylor & Yeeles 2018; Taylor & Yeeles 2019).

Replication fork uncoupling can lead to extensive stretches of RPA-bound ssDNA, which recruits the central replication stress response kinase ATR through its interaction partner ATRIP (MacDougall et al. 2007; Zou & Elledge 2003). Once recruited, ATR gets activated through two specific activator proteins, namely TopBP1 and ETAA1 (Bass et al. 2016; Haahr et al. 2016; Kumagai et al. 2006; Lee et al. 2016). The ssDNA-bound RPA also serves as a recognition site for the Timeless-Tipin complex, which stimulates claspin-mediated phosphorylation of checkpoint kinase 1 (CHK1) by ATR (Kemp et al. 2010). ATR/CHK1 activation leads to fork stabilization and inhibits origin firing, thereby controlling the number of active replisomes. This is important to protect already active forks against irreversible breakage, which can occur upon exhaustion of limiting replication factors such as dNTPs and RPA (Buisson et al. 2015; Toledo et al. 2013). On the other hand, dormant origins in the vicinity of fork-stalling lesions escape from ATR/CHK1-mediated suppression and can rescue stalled forks to ensure completion of DNA synthesis (Ge & Blow 2010; Saldivar et al. 2017).

### Fork protection and restart

To resolve replication stress-inducing problems, multiple mechanisms have evolved to stabilize stalled replication forks and promote their later restart. The choice of fork remodeling and repair pathways is dependent on the type of problem that triggered replication stress as well as the duration of the replication block itself (Berti et al. 2020; Panagopoulos & Altmeyer 2021).

One way to stabilize stalled replication forks is a process known as fork reversal, also referred to as fork regression (Figure 3b). During fork reversal, standard three-way replication forks are converted into four-way junctions by unwinding of the newly synthesized DNA strands and subsequent annealing of the two nascent strands and reannealing of the parental strands (Berti, Cortez, & Lopes 2020). Fork reversal can fine-tune fork speed and pause DNA synthesis in response to a variety of genotoxic treatments, upon oncogene-induced replication stress, and when endogenous replication obstacles are encountered (Berti et al. 2013; Berti, Cortez, & Lopes 2020; Follonier et al. 2013; Neelsen & Lopes 2015; Quinet et al. 2017). After fork uncoupling, RPA-coated ssDNA leads to the recruitment of RAD51, which initiates fork reversal (Bhat & Cortez 2018). Different from the known functions of RAD51 in DNA double-strand break (DSB) repair, RAD51-mediated fork remodeling does not require its classical loading factor BRCA2, nor the formation of stable RAD51 filaments (Mijic et al. 2017; Scully et al. 2019). RAD51-mediated fork reversal is regulated by RADX and the homologous recombination (HR) co-factors RAD51B, RAD51C, RAD51D, XRCC2, and XRCC3 (Berti et al. 2020; Bhat et al. 2018; Halder et al. 2022; Krishnamoorthy et al. 2021). Other proteins involved in fork reversal include SMARCAL1, HLTF, and ZRANB3 (Bai et al. 2020; Kolinjivadi et al. 2017; Poole & Cortez 2017; Taglialatela et al. 2017). SMARCAL1 catalyzes fork regression and Holliday junction migration, thereby promoting efficient fork repair (Bétous et al. 2012). HLTF is a DNA translocase and ubiquitin E3 ligase that gets recruited to the 3’-ssDNA-end of the leading strand where it polyubiquitylates PCNA (Bai et al. 2020; Kile et al. 2015). ZRANB3 interacts with polyubiquitylated PCNA and assists replication fork remodeling through its DNA translocase activity (Ciccia et al. 2012; Vujanovic et al. 2017; Weston et al. 2012). While SMARCAL1 and ZRANB3 guide the initial annealing of the displaced daughter strands, ZRANB3 and HLTF catalyze branch migration (Halder et al. 2022). Upon reversal, the regressed fork resembles a one-ended DSB that must be protected from nucleolytic degradation. Important factors mediating this protection include BRCA1 and RAD51, whose function in fork protection, contrary to the role in initiating fork reversal, is dependent on BRCA2 (Berti, Cortez, & Lopes 2020; Lemacon et al. 2017; Mijic et al. 2017; Tarsounas & Sung 2020).

Stalled and reversed forks can be restarted in multiple ways. Fork restart can be mediated by the helicase RECQ1, which promotes branch migration and converts four-way junctions back into replication-competent three-way junctions. The activity of RECQ1 is regulated by PARP1-dependent poly(ADP-ribosyl)ation (PARylation), which ensures that forks restart only upon repaired damage (Berti et al. 2013). Additionally, re-establishment of three-way junctions can be mediated through fork processing by the Werner syndrome helicase WRN and the DNA2 nuclease (Datta et al. 2021; Thangavel et al. 2015).

Replication obstacles on the leading strand can also be overcome by fork repriming (Bianchi et al. 2013; García-Gómez et al. 2013; Guilliam et al. 2017; Quinet et al. 2021). For repriming, new primers are placed downstream of the obstacles to continue DNA replication, at the cost of leaving behind unreplicated ssDNA gaps that have to be repaired after replication (Mourón et al. 2013). The key enzyme involved in repriming is primase and DNA-directed polymerase (PrimPol), which gets recruited to ssDNA via direct interaction with RPA (González-Acosta et al. 2021; Guilliam et al. 2017). PrimPol not only has primase activity, but also acts as DNA polymerase with low processivity and fidelity (Bianchi et al. 2013; García-Gómez et al. 2013; Guilliam & Doherty 2017; Tirman et al. 2021). Gaps that are left behind the fork can be repaired post-replicatively by either DNA translesion synthesis (TLS), in which specific TLS polymerases mediate replication across the lesion, or by template switching (TS), a process in which the intact sister strand is used for homologous recombination repair (Piberger et al. 2020; Tirman et al. 2021; Wong et al. 2021).

## Fragile sites in the human genome

Certain regions in the genome are particularly vulnerable to endogenous replication stress due to their inherent difficulty to be replicated (Glover et al. 2017; Lezaja & Altmeyer 2021). These difficult-to-replicate regions include chromosomal fragile sites as well as repetitive sequences at ribosomal DNA, centromeres, and telomeres (Figure 3c). Fragile sites are prone to form gaps and breaks visible on metaphase chromosomes, often referred to as fragile site expression (Özer & Hickson 2018). Rare fragile sites are caused by pathological expansion of trinucleotide repeat sequences and are only present in a small percentage of the human population. One example is CGG triplet expansion in the *fragile X messenger ribonucleoprotein 1* (*FMR1*) gene causing fragile X syndrome (Zhou et al. 2016). Common fragile sites (CFSs) on the other hand are present in all individuals, e.g., the fragile sites *FRA16D* and *FRA3B,* which harbor the tumor suppressor genes *fragile histidine triad diadenosine triphosphatase* (*FHIT*) and *WW domain containing oxidoreductase* (*WWOX*), respectively (Durkin & Glover 2007; Özer & Hickson 2018). CFSs in cancer cells are often associated with breakpoints of genomic rearrangements, micro-deletions, and copy number variations (Glover et al. 2017; Sarni & Kerem 2016). The sensitivity of CFSs to replication stress is caused by their tendency to have AT-rich sequences, which are prone to form secondary structures, sparsity of replication origins, association with very long genes that can take more than one cell cycle to be fully transcribed, and their late replication timing, typically being the last regions of the genome to be replicated (Brison et al. 2019; Kaushal et al. 2019). These features challenge faithful and complete replication during S-phase and can lead to under-replicated DNA and CFS instability (Debatisse & Rosselli 2019).

Ribosomal DNA (rDNA) consists of DNA tandem repeats that encode ribosomal RNAs (rRNAs) required for ribosome biosynthesis. Their high rate of transcription makes replication-transcription conflicts almost inevitable. Although replication fork barriers positioned within each rDNA unit were shown to coordinate progression of replication with transcription in eukaryotic cells (Akamatsu & Kobayashi 2015; Gadaleta & Noguchi 2017), R-loops form at transcribed rDNA repeats and cause replication-transcription conflicts in the nucleolus that undermine rDNA stability (El Hage et al. 2010; Lezaja & Altmeyer 2021; Özer & Hickson 2018; Salvi et al. 2014; Tsekrekou et al. 2017; Warmerdam & Wolthuis 2019). Additionally, non-transcribed rDNA repeats cluster in heterochromatic regions at the nucleolar periphery and show, unlike actively transcribed repeats, late replication timing, which makes them prone to form under-replicated DNA and breaks late in the cell cycle (Lezaja & Altmeyer 2021; Warmerdam & Wolthuis 2019).

Centromeres are chromosomal domains needed for faithful transmission of duplicated chromosomes to daughter cells during cell division by assembling the kinetochore and mitotic spindle microtubules for sister chromatid separation. They are composed of a series of 171 nt long AT-rich DNA tandem repeats, named alpha satellites (Barra & Fachinetti 2018). The surrounding pericentromeric heterochromatin is also organized in short tandem repeats. Due to the repetitive nature of centromeric and pericentromeric DNA, secondary structures like DNA loops and catenates are being formed, giving rise to target sites for DNA topoisomerases and the DNA recombination machinery (Barra & Fachinetti 2018). These secondary structures, together with the heterochromatic environment and the late replication timing, contribute to the fragility of centromeric and pericentromeric repeats (Lezaja & Altmeyer 2021; Mitrentsi et al. 2020), and make centromeres hotspots of DNA damage and recombination (Saayman et al. 2023; Yilmaz et al. 2021).

A fourth important class of fragile regions is represented by telomeres, constitutive heterochromatic regions at chromosome ends that determine replicative (im)mortality. Replication stress at telomeres is primarily driven by their composition of terminal tracts of tandem repeats, the presence of secondary DNA structures including G-quadruplexes, R-loops, and telomere loops (t-loops) formed by the G-rich 3’-telomeric ssDNA overhang, and the fact that stalled replication forks downstream of the most distal origin cannot be rescued by dormant origin firing (Lezaja & Altmeyer 2021; Lu & Pickett 2022). Telomeres are protected by the shelterin complex, consisting of the subunits TRF1, TRF2, RAP1, POT1, TPP1, and TIN2 (de Lange 2005). At intact telomeres, the shelterin complex competes with RPA and promotes t-loop formation, thereby suppressing DNA damage response signaling from chromosome ends and unwanted DNA repair reactions that could lead to telomere fusions (Kratz & de Lange 2018). A telomeric long non-coding RNA termed telomeric repeat-containing RNA (TERRA) comprised of G-rich telomere repeats is important for the regulation of telomeric chromatin structure and telomere stability (Azzalin et al. 2007). Binding of TERRA to telomeric DNA leads to the formation R-loops by displacement of the G-rich DNA strand, which are stabilized when telomeres experience elevated levels of replication stress (Feretzaki et al. 2020; Fernandes et al. 2021; Lu & Pickett 2022; Niehrs & Luke 2020).

## Telomere elongation in cancer

**Fig. 4:**
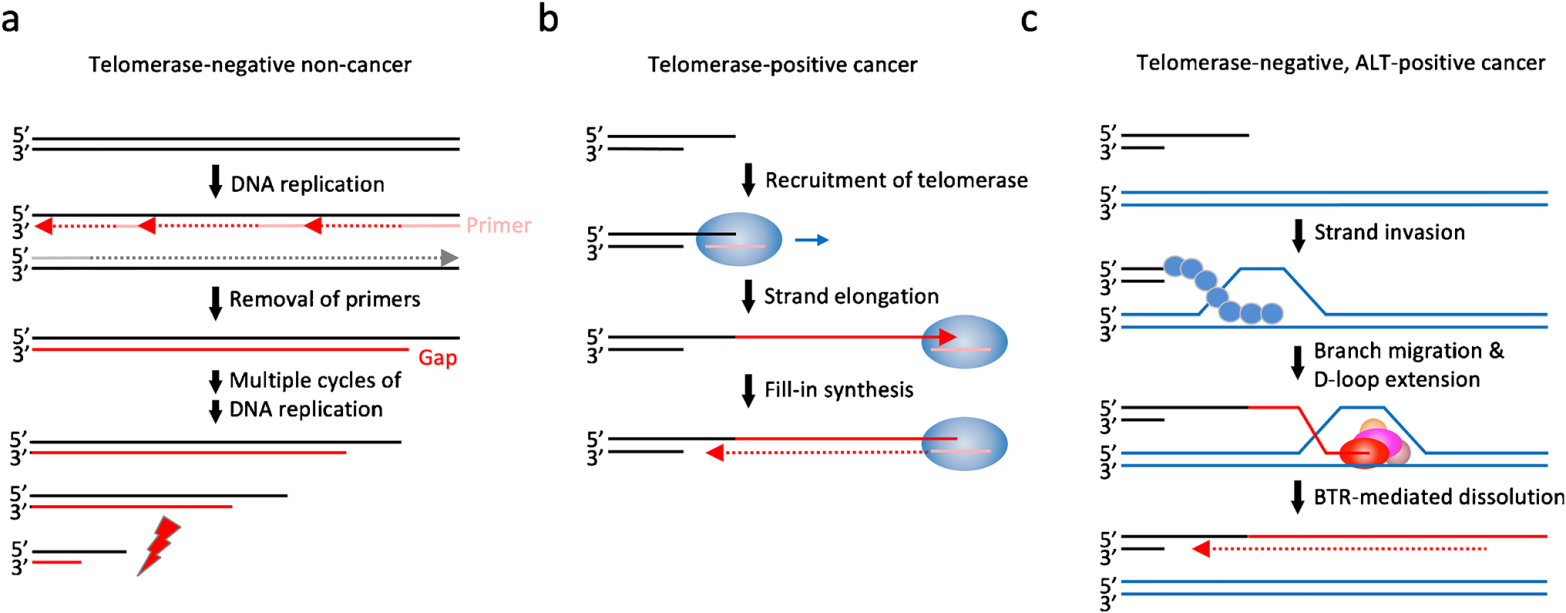
Telomere maintenance in cancer. (a) End-replication problem and successive telomere shortening in somatic cells with inactivated telomerase. Terminal gaps at the lagging strand cause telomere shortening, which can lead to senescence or cell death when telomeres become critically short. (b) Reactivation of telomerase in cancer cells enables replicative immortality. The ribonucleoprotein complex telomerase uses its reverse transcriptase activity and a telomere sequence-containing non-coding RNA for elongation of the telomeric 3’-ssDNA overhang. Successive shortening of telomeres caused by the end-replication problem is countered by telomerase activity. (c) Alternative lengthening of telomeres (ALT) in telomerase-negative cancer cells. ALT-positive cancer cells use recombination-based mechanisms for telomere elongation. For simplicity, productive BTR complex-mediated D-loop dissolution is indicated, although unproductive non-crossover and crossover resolution leading to aborted telomere extension can also occur

Due to the end-replication problem associated with telomeric lagging strand DNA synthesis, telomeres shorten in each cell cycle (Figure 4a). In somatic cells, when critically short telomeres accumulate, cellular senescence, apoptosis, or a permanent cell cycle arrest is triggered (D'Souza et al. 2013; Koliada et al. 2015). In contrast to most somatic cells, stem cells and progenitor cells express low levels of the enzyme telomerase, which extends telomere repeats and contributes to prolonged proliferative capacity. Telomerase is a ribonucleoprotein complex consisting of the enzyme telomerase reverse transcriptase (TERT) and the telomere-sequence containing non-coding human telomerase RNA, which binds to the telomeric 3’-ssDNA overhang (Roake & Artandi 2020). The telomeric 3’-ssDNA overhang is then extended by the reverse transcriptase activity of TERT, using the telomerase RNA as template (Figure 4b). While TERT is usually silenced in somatic cells, most cancer cells show reconstituted expression of the enzyme, thereby achieving replicative immortality (Shay & Wright 2019).

Although expression of telomerase was long seen as a general cancer marker, around 10–15% of tumors, predominantly of mesenchymal origin, lack this enzyme (Bhargava et al. 2022; Lu & Pickett 2022; Shay & Wright 2019). These cancer cells use a homologous recombination-based pathway to extend and maintain their telomeres (Figure 4c), known as alternative lengthening of telomeres (ALT) (Barthel et al. 2017; Dilley & Greenberg 2015; Heaphy et al. 2011). Typically, they display several hallmarks of ALT, including long and heterogeneous telomere length, abundant extrachromosomal circular telomere repeats (ECTR), incorporation of non-telomeric sequences, high levels of telomere sister chromatid exchange, and the formation of ALT-associated PML bodies (APBs) (Bhargava et al. 2022; Bryan et al. 1995; Cesare & Griffith 2004; Londoño-Vallejo et al. 2004; Silva et al. 2019; Yeager et al. 1999a; Zhang & Zou 2020).

APBs are membraneless nuclear condensates that contain the promyelocytic leukemia (PML) protein, clustered telomeres, and several proteins involved in DNA repair, recombination, and replication, thereby generating a local hub for telomere recombination and telomere DNA synthesis (Draskovic et al. 2009; Grobelny et al. 2000; Nabetani et al. 2004; Stavropoulos et al. 2002; Wu et al. 2000; Yeager et al. 1999b). APB formation was suggested to involve phase separation properties of APB-associated proteins, including multivalent SUMO-SIM interactions (Min et al. 2019; Spegg & Altmeyer 2021; Zhang et al. 2020). Of note, the shelterin complex components TRF1 and TRF2 also show features of phase separation and form condensates at telomere repeats (Jack et al. 2022; Soranno et al. 2022). The interplay between TRF1/2-driven telomere condensates and APB condensates in ALT-positive cancer cells remains to be determined.

ALT is induced at stalled and collapsed replication forks, suggesting that replication stress at telomeres and the associated telomere fragility are a driving force behind this process (Amato et al. 2020; Pan et al. 2019; Silva et al. 2021; Turkalo et al. 2023). The ALT pathway was initially characterized in budding yeast mutants lacking functional telomerase, where two distinct subpathways were described: Rad51- and Rad52-mediated amplification of repetitive subtelomeric sequences, and Rad52-dependent but Rad51-independent telomere maintenance by expanding telomeric repeats (Kockler et al. 2021; Le et al. 1999; McEachern & Haber 2006; Zhang & Zou 2020). Also in human cancer cells both RAD51 and RAD52 seem to be involved in ALT-dependent telomere maintenance, either directly or indirectly (Cho et al. 2014; Dilley et al. 2016; Lu & Pickett 2022; Min et al. 2019; Zhang et al. 2019). ALT utilizes break-induced replication (BIR), a recombination process initiated by one-ended DSBs, which results in sequence extension by conservative DNA replication using a homologous template (Anand et al. 2013; Kramara et al. 2018; Zhang & Zou 2020). Resection of one-ended DSBs might be involved, mediated by BLM and DNA2/EXO1 (Nimonkar et al. 2011; Sturzenegger et al. 2014), which can lead to the formation of longer 3’-ssDNA overhangs to facilitate strand invasion of homologous templates. The ssDNA overhang is first bound by RPA before being handed over to RAD52, which promotes annealing of the broken telomere end with a homologous template to form a D-loop (Verma et al. 2019; Zhang et al. 2019). Extension of the D-loop is then mediated by DNA Polδ and its subunits POLD3 and POLD4 (O’Rourke et al. 2019; Zhou et al. 2012). A RAD52-independent ALT pathway seems to exist as well, because RAD52 loss leads to BLM- and POLD3/4-dependent ALT DNA synthesis associated with increased c-circle formation (Epum & Haber 2022; Zhang et al. 2019).

A central positive regulator of ALT is the DNA helicase BLM, which functions in a complex together with TOP3A and RMI1/2, thereby forming the BLM-TOP3A-RMI1/2 (BTR) complex (Bhargava, Lynskey, & O’Sullivan 2022; Manthei & Keck 2013). BLM is critical for ALT-associated DNA synthesis upon telomere clustering, for mitotic DNA synthesis (MiDAS) at telomeres, and for functional APB formation (Min et al. 2019; O'Sullivan et al. 2014; Shorrocks et al. 2021; Sobinoff et al. 2017; Stavropoulos et al. 2002; Zhang et al. 2019). The BLM-containing BTR complex processes recombination intermediates formed during strand invasion and initiates POLD3/4-dependent telomere synthesis. Upon completion of the replicative process, the BTR complex dissolves Holliday junctions, hence its alias *dissolvasome*, thereby preventing the exchange of telomeric sequences between sister chromatids (Sobinoff et al. 2017).

RAD51 associated protein 1 (RAD51AP1) was also shown to be important for telomere clustering and break-induced telomere synthesis (BITS). Loss of RAD51AP1 leads to decreased ALT activity, reduction of APBs, defective recruitment of RAD52 and POLD3 to telomeres, and causes telomere shortening (Barroso-González et al. 2019; Kaminski et al. 2022; Yadav et al. 2022).

Negative regulators of ALT include the ATRX/DAXX histone chaperone complex and the histone variant H3.3, which are often mutated in ALT-positive cancers (Heaphy et al. 2011; Kannan et al. 2012; Minasi et al. 2021; Schwartzentruber et al. 2012). H3.3 mutations deregulate H3K9 methylation and heterochromatin formation at telomeres (Udugama et al. 2022). The ATRX/DAXX histone chaperone complex is involved in H3.3 deposition and chromatin compaction, thereby regulating expression of TERRA and the formation of TERRA R-loops (Bhargava, Lynskey, & O’Sullivan 2022; Clynes et al. 2015; Flynn et al. 2015; Law et al. 2010). The annealing helicase SMARCAL1 was also found to harbor inactivating mutations in ALT-positive cancers (Brosnan-Cashman et al. 2021; Diplas et al. 2018). SMARCAL1 counteracts replication stress at telomeres by promoting fork reversal and fork restart, thereby suppressing ALT (Bétous et al. 2012; Cox et al. 2016; Poole et al. 2015).

The endonuclease SLX4, in complex with SLX1 and ERCC4, plays an opposing role to the BTR complex. While the complex around BLM supports non-crossover dissolution of replication intermediates and productive telomere extension, the complex around SLX4 counteracts dissolution by crossover and non-crossover resolution with aborted telomere extension (Sobinoff et al. 2017). Therefore, a tightly regulated balance between BLM and SLX4 seems to determine ALT productivity and telomere maintenance. An important regulator of this balance is the SLX4 interacting protein SLX4IP. SLX4IP favors SLX4-mediated resolution by antagonizing BLM’s dissolution activity. Loss of SLX4IP leads to an increase in ALT-related phenotypes, and in conjunction with loss of SLX4 to a synthetic growth defect (Panier et al. 2019). While overexpression of SLX4 reduces APB formation, its depletion leads to an increase in APBs, elevated c-circles and ALT telomere extension, and reduces telomeric MiDAS (Özer et al. 2018; Sobinoff et al. 2017). Combined depletion of SLX4 and RAD52 results in increased telomere loss, unresolved telomere recombination intermediates, and mitotic infidelity, representing a synthetic lethal effect (Panier et al. 2019; Verma et al. 2019).

Two proteins of the Fanconi anemia pathway were also shown to control ALT activity: FANCD2 counteracts BLM-mediated resection and strand exchange, which promotes intramolecular resolution of stalled replication forks during ALT. Loss of FANCD2, similar to the loss of SLX4IP, leads to hyperactivation of ALT with increased extrachromosomal telomeric DNA and recombinational byproducts (Root et al. 2016). The ATPase and DNA translocase FANCM controls ALT at multiple levels: Similar to SMARCAL1, FANCM promotes remodeling of stalled replication forks and fork reversal, thereby counteracting replication stress at telomeres (Gari et al. 2008). FANCM also counteracts replication stress prior to fork stalling by controlling TERRA levels and regulating telomeric R-loop formation (Silva et al. 2019). Additionally, FANCM interacts with the BTR complex and regulates its branch migration activity (Lu et al. 2019; Silva et al. 2019).

Telomeric DNA synthesis during ALT occurs in S/G2 and in mitosis, indicating that telomere elongation and maintenance are not completed during S-phase. Recent findings suggest that telomere replication and recombination intermediates in ALT-positive cancer cells are even transmitted to the next cell cycle, where they are shielded by RPA to prevent excessive telomere damage and promote a process termed post-mitotic DNA synthesis (post-MiDAS) in G1 cells (Lezaja et al. 2021). Thus, telomere maintenance by ALT seems uncoupled from the general cell cycle-embedded principle of temporally separating genome duplication and maintenance from cell division. Clustering of telomere repeats from multiple chromosomes may allow telomere recombination irrespective of cell cycle phase, and in the next paragraphs we discuss emerging principles of telomere clustering in ALT-positive cancer cells in connection with RPA condensation.

## DNA repair condensates and ALT

Biomolecular condensates that support DNA repair reactions, their molecular compositions, mechanisms of assembly, and functions are manifold (Alghoul et al. 2023; Dall'Agnese et al. 2023; Laflamme & Mekhail 2020; Mine-Hattab et al. 2022; Spegg & Altmeyer 2021). Their formation typically follows a multi-step process, in which several types of associative interactions cooperate to build functional compartments (Spegg & Altmeyer 2021). This multi-step process, once initiated through specific interactions at sites of DNA damage, can be seen as a self-perpetuating assembly process, promoted in part by self-association of the recruiting factors. In addition to such feed-forward amplification, negative feedback regulation is typically also involved to avoid excessive (in space and/or time) recruitment (Altmeyer & Lukas 2013). Interestingly, ALT itself is a self-perpetuating process: ALT activity promotes replication stress, which in turn induces a BIR-driven feedforward loop of SUMO-dependent repair protein recruitment and ALT telomere synthesis in APB condensates (Zhang et al. 2021). Disruption of this feedforward loop results in reduced replication stress at telomeres and reduced RPA recruitment. RPA protects telomeric ssDNA in ALT-positive cancer cells not only in S/G2 but also at post-MiDAS sites in G1 (Lezaja et al. 2021). Despite its ultra-high affinity for ssDNA, the RPA complex readily phase separates in solution through associative interactions to form ssDNA-containing liquid droplets (Spegg et al. 2023). An excess of free RPA was previously shown to facilitate rapid exchange of RPA on ssDNA (Gibb et al. 2014; Ma et al. 2016). Consistently, sub-stoichiometric amounts of ssDNA were most effective in triggering dynamic RPA condensates (Spegg et al. 2023). Taken together, these findings suggest that RPA condensation generates a reservoir of highly concentrated RPA around ssDNA to promote rapid exchange between the free and bound state and allow handover to downstream ssDNA-binding proteins such as RAD51/RAD52 (Spegg et al. 2023). This model implies non-stoichiometric assembly of RPA on ssDNA, with the surplus of RPA around ssDNA facilitating continuous RPA exchange. RPA condensation properties are modulated by phosphorylation-induced negative charges on an intrinsically disordered region (IDR), and phosphomimetic mutants of RPA fail to form liquid droplets in vitro and light-induced condensates in cells (Spegg et al. 2023). Charge blockiness, rather than specific target site phosphorylation, was recently shown to regulate cell cycle-specific phase separation (Yamazaki et al. 2022) and, consistently, multisite phosphorylation of the IDR in RPA cooperatively affects RPA clustering (Spegg et al. 2023). Cells expressing phosphomimetic RPA show altered ALT activity, with reduced telomere clustering, elevated ssDNA at telomeres, impaired RAD52 recruitment, and increased telomere loss (Spegg et al. 2023). As telomere clustering is a hallmark of ALT, defective clustering may cause unproductive telomere synthesis and exacerbated telomeric DNA damage. Although the exact mechanism of impaired telomere clustering in RPA phosphomimetic mutant cells remains to be fully elucidated, several observations suggest an emerging connection between RPA condensation at fragile genomic regions, including ALT telomeres, and activation of the nuclear cytoskeleton for enhanced chromatin mobilization.

## Emerging links between repair condensates and the nuclear cytoskeleton

Using optogenetic tools for controlled light-inducible Cry2-dependent protein condensation (Kilic et al. 2019; Shin et al. 2017) coupled to sensitive TurboID proximity labeling proteomics (Alghoul et al. 2021; Frattini et al. 2021) revealed that RPA condensation not only results in selective partitioning of RAD52 and the ALT-promoting BTR complex, but also in the selective enrichment of several components of the actin- and myosin network (Spegg et al. 2023). Considering that the light-induced clustering of RPA and the simultaneous TurboID-mediated proximity labeling were performed for a comparatively short duration of only 15 minutes, the identified proteins likely represent the first responders to RPA condensation. Among them were ACTN4, MYO1C, and MYH9, which have known nuclear functions in chromatin organization, transcription, and post-mitotic nuclear expansion (Almuzzaini et al. 2015; Krippner et al. 2020; Kumeta et al. 2010; Sarshad et al. 2013; Ye et al. 2020). As central component of the cytoskeleton, actin plays fundamental roles in cell division, cell movement, cell signaling, and organelle transport across species (Boldogh et al. 2001; Chakrabarti et al. 2018; Grosse et al. 2003). Actin exists in a monomeric globular form (G-actin) or as polymerized multimers forming the filamentous actin (F-actin) network (Dominguez & Holmes 2011; Gunning et al. 2015). Myosins, on the other hand, are ATP-dependent motor proteins that move along actin filaments and transport cargo (Minozzo & Rassier 2013; Woolner & Bement 2009).

The actinomyosin network is not only involved in cytoplasmic transport processes, but was more recently also shown to participate in nuclear processes such as chromatin decondensation and nuclear volume expansion after cell division, initiation of DNA replication, and in the regulation of transcription by enhancing RNA polymerase II clustering (Baarlink et al. 2017; Krippner et al. 2020; Parisis et al. 2017; Plessner & Grosse 2019; Ulferts et al. 2021; Wei et al. 2020). Intriguingly, nuclear actin filaments were also shown to form upon treatment with different DNA damaging agents including UV-radiation, methylmethanosulfonate (MMS), and neocarzinostatin (NCS), and are increasingly recognized to play a role in DNA repair (Andrin et al. 2012; Belin et al. 2015; Hurst et al. 2019). The actin regulating ARP2/3 protein complex and its associated factor WASP were found to localize to sites of DNA damage in mammalian cells where they nucleate actin filament formation. This promotes DSB mobility and clustering for repair by HR (Schrank et al. 2018; Schrank & Gautier 2019). Consistently, nuclear actin polymerization and myosin are required for the directed movement of DSBs within heterochromatin towards the nuclear periphery for error-free HR repair in Drosophila and mammalian cells (Caridi et al. 2018; Caridi et al. 2019; Merigliano & Chiolo 2021; Rawal et al. 2019), and damaged rDNA relocalizes to the nucleolar periphery in an ATM-, ARP2/3-, and myosin-dependent manner (Harding et al. 2015; Marnef et al. 2019). Similarly, rDNA breaks in yeast transiently move to extranucleolar regions for recombinational repair (Torres-Rosell et al. 2007), and breaks in pericentric heterochromatin of mouse cells relocate to the periphery of heterochromatin domains after resection (Tsouroula et al. 2016). More recently, nuclear F-actin was also found to play a role in response to replication stress in mammalian cells, where it counteracts nuclear deformation and promotes myosin-dependent re-localization of stressed replication forks to the nuclear periphery in an ATR- and WASP-ARP2/3-dependent manner (Lamm et al. 2020; Lamm et al. 2021). Interestingly, WASP associates with RPA at stressed replication forks and promotes RPA binding to ssDNA (Han et al. 2022), and actin nucleators regulate RPA availability under conditions of replication stress (Nieminuszczy 2023).

**Fig. 5:**
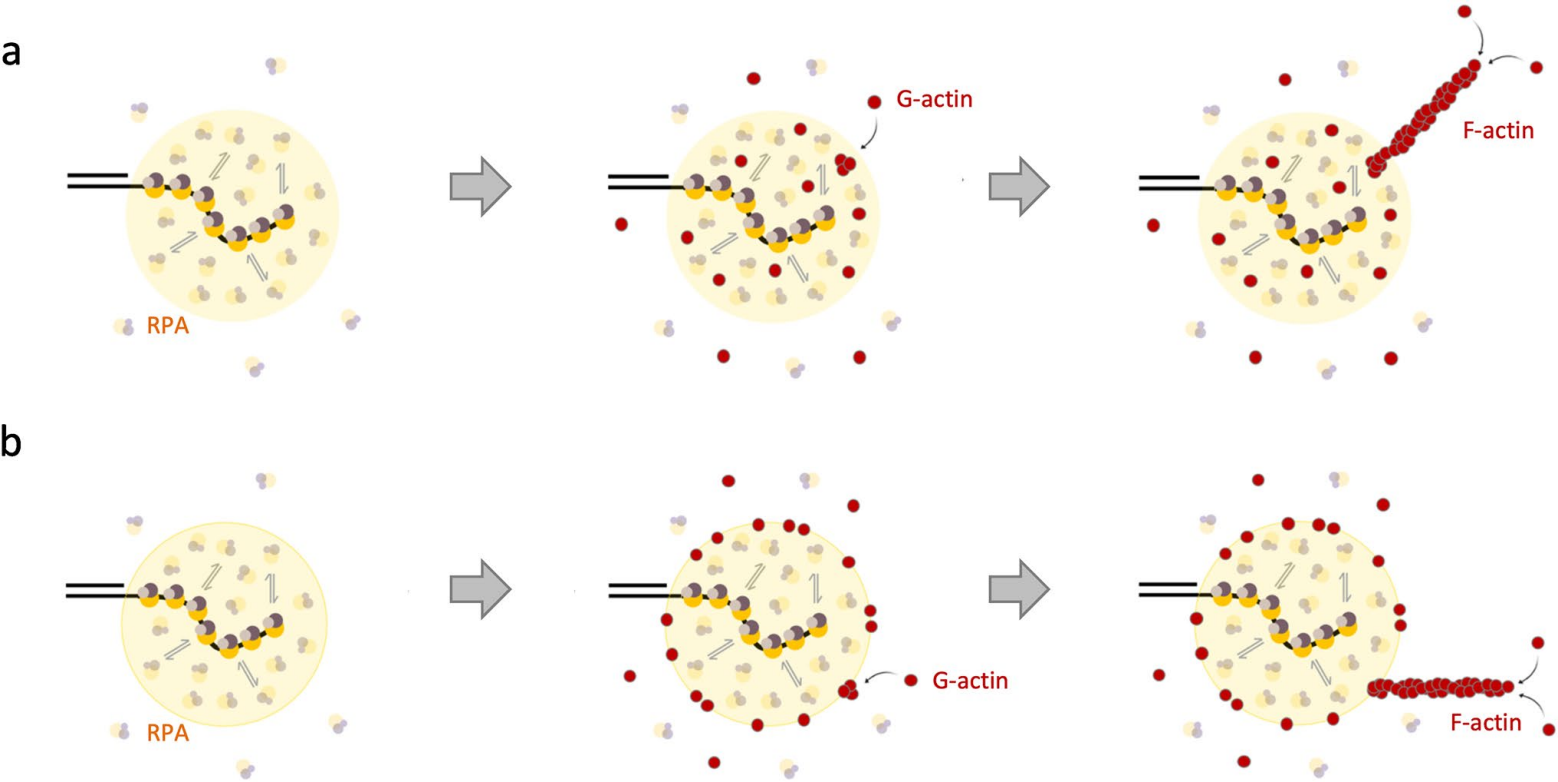
Emerging links between biomolecular condensates and activation of the nucleoskeleton. (a) Model of actin filament formation through G-actin concentration in DNA repair condensates formed by RPA and associated proteins. (b) Model of actin filament formation through G-actin concentration on the surface of DNA repair condensates formed by RPA and associated proteins

Reciprocally, RPA condensates may concentrate monomeric G-actin and thereby trigger nucleation and growth of actin filaments at sites of DNA damage (Figure 5a). Previous work has shown that self-assembled polypeptide condensates serve as hub for actin enrichment and polymerization in vitro (Graham et al. 2023; McCall et al. 2018). Furthermore, condensation of actin by intrinsically disordered regions of actin-associated proteins was proposed as general mechanism for actin network organization (Billault-Chaumartin et al. 2022). Consistently, during oocyte development, actomyosin cortex activation is promoted by the emergence of thousands of short-lived protein condensates enriched in actin, WASP, and ARP2/3 forming an active micro-emulsion (Yan et al. 2022). Phase separation of actin regulatory factors was shown to increase the dwell time of nucleators to initiate F-actin formation, as demonstrated for WASP and ARP2/3 at the cell membrane (Case et al. 2019). Reducing dynamic interaction landscapes from a 3D environment to a 2D interface may generally help to concentrate molecules and promote their activation. Along these lines, actin and actin nucleation factors might get selectively enriched on the surface of RPA condensates through interfacial affinity, rather than in the interior (Figure 5b). Similar interactions have been observed between microtubule subunits and stress granules (Böddeker et al. 2022).

**Fig. 6:**
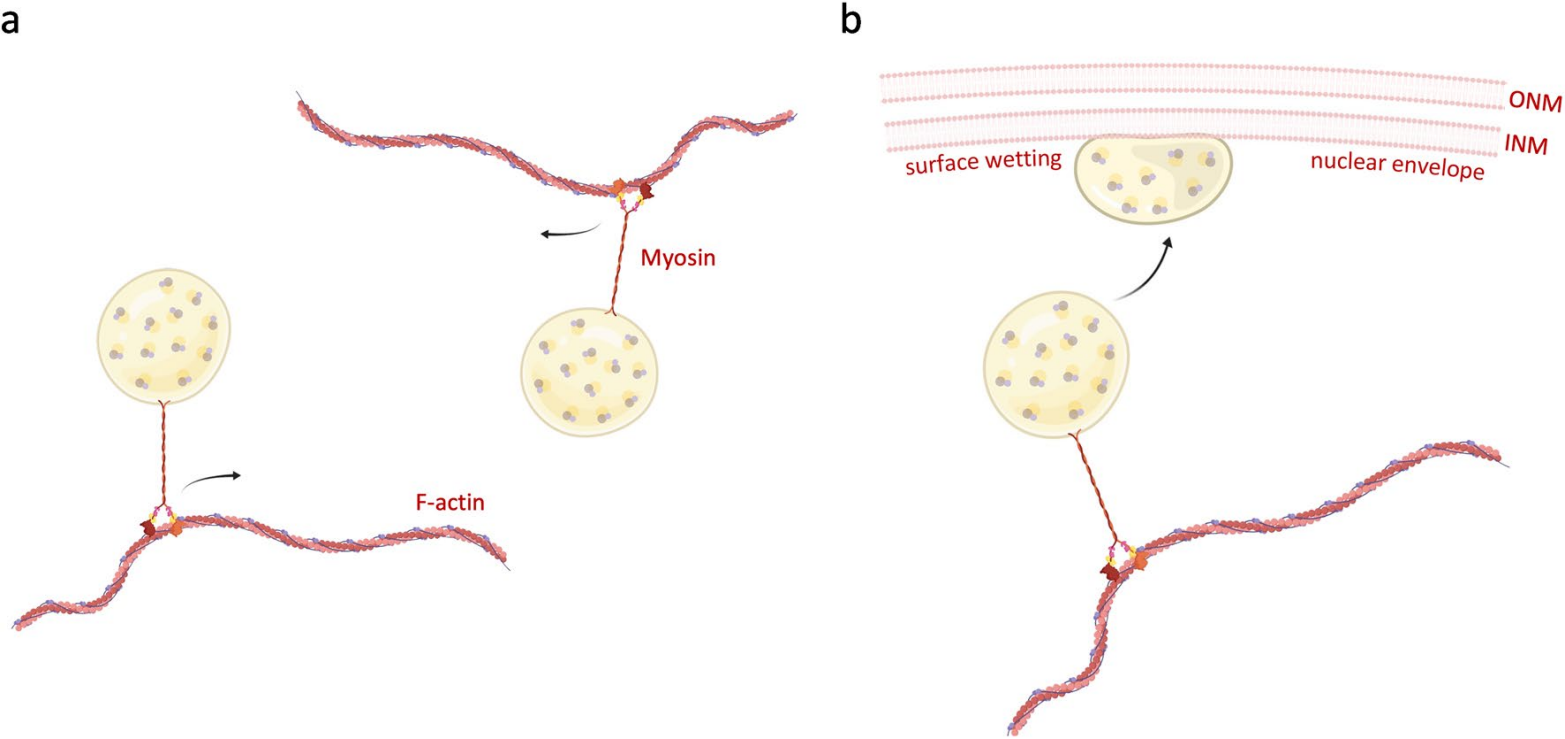
DNA repair condensate mobilization and surface wetting at the nuclear envelope. (a) Model of actinomyosin-mediated movement of DNA repair condensates through the nuclear space. (b) Model of surface wetting-assisted anchoring of DNA repair condensates at the nuclear periphery. INM, inner nuclear membrane; ONM, outer nuclear membrane

In yeast, Rad52-dependent DNA repair condensates were previously shown to induce nuclear microtubule filaments, which is required for moving the damaged DNA compartment to the nuclear periphery for repair (Oshidari et al. 2020). Microtubule-dependent DNA damage mobility was also observed in mouse cells with unprotected, dysfunctional telomeres (Lottersberger et al. 2015). In human cancer cells that use ALT-dependent recombination at fragile telomeres, nuclear actin filaments might serve as molecular highways for the directed movement of RPA-enriched repair condensates, with the condensate surface or co-condensing adaptor molecules serving as anchoring points for myosin (Figure 6a). A conceptually related mechanism seems to be at work during neuronal long-distance transport of RNAs, where phase-separated RNA granules hitchhike on lysosomes through a low complexity domain-containing tether protein (Liao et al. 2019).

Both in yeast and in human cells, stressed telomeres relocalize to the nuclear periphery and this process is driven by nuclear F-actin polymerization and involves RPA and SUMOylation (Churikov et al. 2016; Pinzaru et al. 2020; Spichal et al. 2016). Interestingly, RPA contains a SIM motif, through which it can interact with SUMOylated proteins (Zhu et al. 2023), and RPA itself is SUMOylated when collapsed replication forks are relocated to the nuclear periphery in yeast (Whalen et al. 2020). Whether RPA SUMOylation and RPA-SUMO interactions are involved in RPA condensation and actin polymerization remains to be addressed.

Similar to actin, also myosin is increasingly recognized to play important roles in the nucleus for transcription and in the DNA damage response, and was recently shown to regulate phase separation by promoting condensate coalescence (Cook & Toseland 2021; Feng et al. 2022; Hari-Gupta et al. 2022). The actinomyosin network appears to be more important for homology-directed repair and RAD52-mediated single-strand annealing (SSA), compared to DNA repair by non-homologous end-joining (NHEJ) or alternative end-joining (alt-EJ) (Pfitzer et al. 2019), consistent with a predominant role during homology-directed repair of fragile repetitive sequences such as telomeres. Moreover, formation of nuclear F-actin in response to genotoxic stress was recently shown to serve as scaffold for PML nuclear bodies (Cobb et al. 2022). Whether the same holds true for APBs at telomeres remains to be shown. Nevertheless, several connections between self-assembly features of repair condensates and the nuclear cytoskeleton have started to emerge, spurring considerations about the mechanobiology of genome maintenance.

## Connecting DNA repair condensates to mechanobiology

Cells are exposed to dynamically changing extrinsic mechanical forces, including shear stress, stretching, stiffening, and compression, and these forces are counteracted by cell-intrinsic force generation through the cytoskeleton and through modulation of the viscoelastic properties of the cellular interior (Bertillot et al. 2022; Dupont & Wickström 2022). With the intensified research on material properties of biomolecular condensates, new connections between mechanobiology and viscoelastic polymer networks in membraneless organelles are being revealed (Lee et al. 2022; Wiegand & Hyman 2020). For instance, recent work demonstrated that cytoplasmic forces tune nuclear condensate coalescence and molecular kinetics within condensates (Al Jord et al. 2022). Mechanical force experienced by the nucleus modulates permeability across nuclear pores, indicating that transport of proteins through the hydrogel-like meshwork of FG-rich repeats inside nuclear pore complexes is mechanosensitive (Andreu et al. 2022). Moreover, mechanosensing of cell density by adhesion forces was shown to control cell cycle progression at the G2/M transition through activation of the WEE1 checkpoint kinase (Donker et al. 2022). Alterations in cell tension also affects cell cycle progression from G1 to S-phase (Perez-Gonzalez et al. 2019), and the mechanosensing Hippo pathway with its mechanotransducers YAP/TAZ and LATS1/2 has also been implicated in DNA damage signaling and repair (Pefani et al. 2014; Pefani & O'Neill 2016).

Nuclear condensates are not only scaffolds for biochemical reactions, but also mechanic entities with viscoelastic properties that generate and respond to mechanical force (Spagnol et al. 2016). Similarly, the chromatin polymer itself is a main factor in defining nuclear mechanical properties (Barbieri et al. 2013; Nicodemi & Pombo 2014; Stephens et al. 2019). Depending on the time and length scale, chromatin behaves both as an elastic solid and as a viscous liquid (Zidovska 2020), a rheological behavior that is also observed for the cytoskeleton (Wiegand & Hyman 2020). The viscoelastic properties of the nuclear environment is regulated by several factors, including external cytoskeletal forces that change nuclear morphology, the nuclear lamina, chromatin compaction and structural organization, and the formation and regulation of biomolecular condensates (dos Santos & Toseland 2021). As physical properties and dynamic forces are often altered in human disease, which can deregulate gene expression programs and challenge genome stability, understanding the interplay between genome organization and mechanobiology may have clinical implications.

Nuclear deformation in migrating immune and metastatic cancer cells causes nuclear blebbing and rupture and is associated with increased DNA damage, depletion of DNA repair factors, and cell cycle checkpoint activation (Denais et al. 2016; Irianto et al. 2016; Irianto et al. 2017; Isermann & Lammerding 2017; Pfeifer et al. 2018; Raab et al. 2016; Xia et al. 2018; Xia et al. 2019; Xie et al. 2020). Moreover, external mechanical stimuli from the cytoplasm can modulate processes inside the nucleus by transmitting force through connections between the cytoskeleton and the nucleoskeleton (Dupont & Wickström 2022; Goelzer et al. 2021). Such connections are provided by linker of nucleoskeleton and cytoskeleton (LINC) complexes (Alam et al. 2016; Crisp et al. 2005; Leno 1992; Mammoto et al. 2012; Wang et al. 2018). LINC complexes, embedded in the nuclear envelope, connect cytoplasmic intermediary filaments, microtubules, and actin filaments with the nuclear lamina and with silenced heterochromatin regions in lamina-associated domains (LADs) (dos Santos & Toseland 2021; Spichal & Fabre 2017). Defects in the nuclear lamina are associated with diseases like Hutchinson-Gilford progeria, muscular dystrophy, and cardiomyopathies, and can lead to changes in chromatin structure as well as deregulated DNA replication, repair, and gene expression (Cho et al. 2019; dos Santos & Toseland 2021; Schreiber & Kennedy 2013). Interestingly, LINC complex components are involved in DNA damage relocalization and clustering and promote homologous recombination repair (Aymard et al. 2017; Bozec et al. 2023; Lawrence et al. 2016; Lottersberger et al. 2015; Marnef et al. 2019; Swartz et al. 2014). Moreover, the LINC complex proteins SUN1 and SUN2, together with dynamic microtubules and nuclear pore proteins, drive the formation of DSB-capturing nuclear envelope tubules (dsbNETs) to support repair in the interior of the nucleus (Shokrollahi et al. 2023).

Condensed chromatin is a barrier for the DNA repair machinery (Mitrentsi et al. 2022), and the local viscoelasticity of the nucleus varies by compartment and degree of chromatin condensation (Lee et al. 2022). While nucleosomes and nucleosome clusters on the nanoscale are mobile and have liquid-/gel-like properties, condensed chromatin polymers on the mesoscale seem physically constrained and more immobile, with soluble chromatin-binding proteins coalescing on the solid chromatin scaffold (Hansen et al. 2021; Strickfaden et al. 2020; Tortora et al. 2022). Chromatin decompaction is needed to improve the efficiency of DNA repair upon damage (Polo & Almouzni 2015), and induced chromatin decompaction reduces nuclear stiffness by ~35–50% (Hobson et al. 2020; Krause et al. 2013; Shimamoto et al. 2017; Stephens et al. 2017). The change from a locally stiffer to a softer chromatin environment may energetically favor nucleation and growth of DNA repair condensates. Nuclear stiffness is indeed reduced upon DNA damage and the reduction in nuclear tension promotes repair (dos Santos et al. 2021). Moreover, nuclear softening upon severe nuclear deformation, driven by Piezo1-triggered reduction of lamina-associated H3K9me3-marked heterochromatin to insulate the genetic material from mechanical force, promotes genome stability (Nava et al. 2020).

The size to which biomolecular condensates can grow in a viscoelastic environment is limited, because with increasing growth more energy is required to deform the surrounding stiffer matrix (Lee et al. 2022; Wiegand & Hyman 2020). Indeed, the chromatin polymer may mechanically suppress droplet coalescence and ripening and control condensate number, size, and positioning (Zhang et al. 2021). Such considerations could also apply to DNA repair condensates and might affect their mobility and growth. Possibly, repair condensates redirected to the nuclear periphery for error-free repair could wetten the inner nuclear membrane (Mangiarotti et al. 2022; Oshidari et al. 2020; Strom et al. 2023), which might be a mechanism to anchor repair compartments to the nuclear envelope by means of adsorption (Figure 6b). Upon membrane wetting, biochemical reactions at the 2D membrane-condensate interface may be accelerated compared to the 3D volume of a non-membrane-tethered condensate, potentially providing an additional advantage for genome repair.

## Conclusions and perspectives

Recent work has started to unveil intriguing connections between nuclear condensates involved in the cellular response to replication stress and DNA damage and the nuclear cytoskeleton. Moreover, material properties and the mechanobiology of chromatin, nuclear condensates, and the nuclear cytoskeleton are receiving increasing attention. While it is becoming clear that genome functions and cellular responses to stress, including genotoxic stress, are tightly linked to nuclear architecture and to the dynamically changing material properties of the nuclear interior, the varying length- and timescales at which different nuclear processes and macromolecular assemblies occur complicate their analysis and interpretation. Many open questions remain about whether and how nuclear condensates sense mechanical stimuli and if they regulate genome functions (e.g. chromatin organization, epigenetic states, replication timing, DNA repair) in response to external forces. Conversely, how changes in chromatin structure and genome stability affect nuclear mechanobiology is currently not well understood.

Fragile telomeres, which per se exhibit strong subdiffusive motion (Lee et al. 2022) yet become mobilized upon telomeric replication stress and DNA damage (Lamm et al. 2021), may represent a paradigm for emerging connections between viscoelastic repair condensates formed around damaged genomic regions and nuclear mechanobiology. Further insights into the material properties of chromatin domains and nuclear compartments and how they are linked to material properties of the cytoskeleton and of the surrounding membranes are going to benefit a mechanistic understanding of genome functioning and its deregulation in disease. Elucidating the interplay between material properties and biochemical reactions in cells may also enable their targeted modulation, e.g., by shifting material properties from liquid-/gel-like to stiffening, and vice versa, or by inducing local stirring of molecules. Considering that material properties of cellular components, including chromatin compartments, biomolecular condensates in- and outside the nucleus, membranes, and the cytoskeleton, may age and experience fatigue in diseases such as cancer and neurodegeneration, integrating concepts from soft matter physics and polymer mechanics, from material science and engineering, and from theoretical modelling and computer simulations, may reveal new biology and open new avenues for biomedical research.

## References

[CR1] Akamatsu Y, Kobayashi T (2015). The human RNA polymerase I transcription terminator complex acts as a replication fork barrier that coordinates the progress of replication with rRNA transcription activity. Mol Cell Biol.

[CR2] Al Jord A, Letort G, Chanet S, Tsai FC, Antoniewski C, Eichmuller A, Da Silva C, Huynh JR, Gov NS, Voituriez R (2022). Cytoplasmic forces functionally reorganize nuclear condensates in oocytes. Nat Commun.

[CR3] Alam SG, Zhang Q, Prasad N, Li Y, Chamala S, Kuchibhotla R, Kc B, Aggarwal V, Shrestha S, Jones AL (2016). The mammalian LINC complex regulates genome transcriptional responses to substrate rigidity. Sci Rep.

[CR4] Alghoul E, Basbous J, Constantinou A (2021). An optogenetic proximity labeling approach to probe the composition of inducible biomolecular condensates in cultured cells. STAR Protoc.

[CR5] Alghoul E, Basbous J, Constantinou A (2023). Compartmentalization of the DNA damage response: mechanisms and functions. DNA Repair.

[CR6] Almuzzaini B, Sarshad AA, Farrants AK, Percipalle P (2015). Nuclear myosin 1 contributes to a chromatin landscape compatible with RNA polymerase II transcription activation. BMC Biol.

[CR7] Altmeyer M, Lukas J (2013). Guarding against collateral damage during chromatin transactions. Cell.

[CR8] Amato R, Valenzuela M, Berardinelli F, Salvati E, Maresca C, Leone S, Antoccia A, Sgura A (2020). G-quadruplex stabilization fuels the ALT pathway in ALT-positive osteosarcoma cells. Genes.

[CR9] Anand RP, Lovett ST, Haber JE (2013). Break-induced DNA replication. Cold Spring Harb Perspect Biol.

[CR10] Andreu I, Granero-Moya I, Chahare NR, Clein K, Molina-Jordan M, Beedle AEM, Elosegui-Artola A, Abenza JF, Rossetti L, Trepat X (2022). Mechanical force application to the nucleus regulates nucleocytoplasmic transport. Nat Cell Biol.

[CR11] Andrin C, McDonald D, Attwood KM, Rodrigue A, Ghosh S, Mirzayans R, Masson JY, Dellaire G, Hendzel MJ (2012). A requirement for polymerized actin in DNA double-strand break repair. Nucleus.

[CR12] Arezi B, Kuchta RD (2000). Eukaryotic DNA primase. Trends Biochem Sci.

[CR13] Aymard F, Aguirrebengoa M, Guillou E, Javierre BM, Bugler B, Arnould C, Rocher V, Iacovoni JS, Biernacka A, Skrzypczak M (2017). Genome-wide mapping of long-range contacts unveils clustering of DNA double-strand breaks at damaged active genes. Nat Struct Mol Biol.

[CR14] Azzalin CM, Reichenbach P, Khoriauli L, Giulotto E, Lingner J (2007). Telomeric repeat–containing RNA and RNA surveillance factors at mammalian chromosome ends. Science.

[CR15] Baarlink C, Plessner M, Sherrard A, Morita K, Misu S, Virant D, Kleinschnitz EM, Harniman R, Alibhai D, Baumeister S (2017). A transient pool of nuclear F-actin at mitotic exit controls chromatin organization. Nat Cell Biol.

[CR16] Bai G, Kermi C, Stoy H, Schiltz CJ, Bacal J, Zaino AM, Hadden MK, Eichman BF, Lopes M, Cimprich KA (2020). HLTF promotes fork reversal, limiting replication stress resistance and preventing multiple mechanisms of unrestrained DNA synthesis. Mol Cell.

[CR17] Barbieri M, Scialdone A, Piccolo A, Chiariello AM, di Lanno C, Prisco A, Pombo A, Nicodemi M (2013). Polymer models of chromatin organization. Front Genet.

[CR18] Barnum KJ, O’Connell MJ, Noguchi E, Gadaleta MC (2014). Cell cycle regulation by checkpoints. Cell cycle control: mechanisms and protocols.

[CR19] Barra V, Fachinetti D (2018). The dark side of centromeres: types, causes and consequences of structural abnormalities implicating centromeric DNA. Nat Commun.

[CR20] Barroso-González J, García-Expósito L, Hoang SM, Lynskey ML, Roncaioli JL, Ghosh A, Wallace CT, Modesti M, Bernstein KA, Sarkar SN (2019). RAD51AP1 is an essential mediator of alternative lengthening of telomeres. Mol Cell.

[CR21] Barthel FP, Wei W, Tang M, Martinez-Ledesma E, Hu X, Amin SB, Akdemir KC, Seth S, Song X, Wang Q (2017). Systematic analysis of telomere length and somatic alterations in 31 cancer types. Nat Genet.

[CR22] Bass TE, Luzwick JW, Kavanaugh G, Carroll C, Dungrawala H, Glick GG, Feldkamp MD, Putney R, Chazin WJ, Cortez D (2016). ETAA1 acts at stalled replication forks to maintain genome integrity. Nat Cell Biol.

[CR23] Basu S, Greenwood J, Jones AW, Nurse P (2022). Core control principles of the eukaryotic cell cycle. Nature.

[CR24] Belin BJ, Lee T, Mullins RD (2015). DNA damage induces nuclear actin filament assembly by Formin-2 and Spire-1/2 that promotes efficient DNA repair. eLife.

[CR25] Berti M, Cortez D, Lopes M (2020). The plasticity of DNA replication forks in response to clinically relevant genotoxic stress. Nat Rev Mol Cell Biol.

[CR26] Berti M, Ray Chaudhuri A, Thangavel S, Gomathinayagam S, Kenig S, Vujanovic M, Odreman F, Glatter T, Graziano S, Mendoza-Maldonado R (2013). Human RECQ1 promotes restart of replication forks reversed by DNA topoisomerase I inhibition. Nat Struct Mol Biol.

[CR27] Berti M, Teloni F, Mijic S, Ursich S, Fuchs J, Palumbieri MD, Krietsch J, Schmid JA, Garcin EB, Gon S (2020). Sequential role of RAD51 paralog complexes in replication fork remodeling and restart. Nat Commun.

[CR28] Berti M, Vindigni A (2016). Replication stress: getting back on track. Nat Struct Mol Biol.

[CR29] Bertillot F, Miroshnikova YA, Wickstrom SA (2022). SnapShot: Mechanotransduction in the nucleus. Cell.

[CR30] Bétous R, Mason AC, Rambo RP, Bansbach CE, Badu-Nkansah A, Sirbu BM, Eichman BF, Cortez D (2012). SMARCAL1 catalyzes fork regression and Holliday junction migration to maintain genome stability during DNA replication. Genes Dev.

[CR31] Bhargava R, Lynskey ML, O’Sullivan RJ (2022). New twists to the ALTernative endings at telomeres. DNA Repair.

[CR32] Bhat KP, Cortez D (2018). RPA and RAD51: fork reversal, fork protection, and genome stability. Nat Struct Mol Biol.

[CR33] Bhat KP, Krishnamoorthy A, Dungrawala H, Garcin EB, Modesti M, Cortez D (2018). RADX modulates RAD51 activity to control replication fork protection. Cell Rep.

[CR34] Bianchi J, Rudd SG, Jozwiakowski SK, Bailey LJ, Soura V, Taylor E, Stevanovic I, Green AJ, Stracker TH, Lindsay HD (2013). PrimPol bypasses UV photoproducts during eukaryotic chromosomal DNA replication. Mol Cell.

[CR35] Billault-Chaumartin I, Muriel O, Michon L, Martin SG (2022) Condensation of the fusion focus by the intrinsically disordered region of the formin Fus1 is essential for cell-cell fusion. Curr Biol 32(21):4752–4761 10.1016/j.cub.2022.09.026 PMC9671092 36202103

[CR36] Bleichert F (2019). Mechanisms of replication origin licensing: a structural perspective. Curr Opin Struct Biol.

[CR37] Blow JJ, Ge XQ, Jackson DA (2011). How dormant origins promote complete genome replication. Trends Biochem Sci.

[CR38] Blow JJ, Gillespie PJ (2008). Replication licensing and cancer—a fatal entanglement?. Nat Rev Cancer.

[CR39] Böddeker TJ, Rosowski KA, Berchtold D, Emmanouilidis L, Han Y, Allain FHT, Style RW, Pelkmans L, Dufresne ER (2022). Non-specific adhesive forces between filaments and membraneless organelles. Nat Phys.

[CR40] Boldogh IR, Yang HC, Nowakowski WD, Karmon SL, Hays LG, Yates JR, Pon LA (2001). Arp2/3 complex and actin dynamics are required for actin-based mitochondrial motility in yeast. Proc Natl Acad Sci U S A.

[CR41] Boos D, Ferreira P (2019). Origin firing regulations to control genome replication timing. Genes.

[CR42] Bozec BL, Guitton-Sert L, Collins S, Rocher V, Guillou E, Payrault C, Arnould C, Guénolé A, Aguirrebengoa M, Finoux A-L et al (2023) Circadian PERIOD complex regulates TC-DSB repair through anchoring to the nuclear envelope. bioRxiv https://www.biorxiv.org/content/10.1101/2023.05.11.540338v1 10.1016/j.molcel.2025.10.027 41270757

[CR43] Brickner JR, Garzon JL, Cimprich KA (2022). Walking a tightrope: the complex balancing act of R-loops in genome stability. Mol Cell.

[CR44] Brison O, El-Hilali S, Azar D, Koundrioukoff S, Schmidt M, Nähse V, Jaszczyszyn Y, Lachages A-M, Dutrillaux B, Thermes C (2019). Transcription-mediated organization of the replication initiation program across large genes sets common fragile sites genome-wide. Nat Commun.

[CR45] Brosnan-Cashman JA, Davis CM, Diplas BH, Meeker AK, Rodriguez FJ, Heaphy CM (2021). SMARCAL1 loss and alternative lengthening of telomeres (ALT) are enriched in giant cell glioblastoma. Mod Pathol.

[CR46] Bryan TM, Englezou A, Gupta J, Bacchetti S, Reddel R (1995). Telomere elongation in immortal human cells without detectable telomerase activity. EMBO J.

[CR47] Buisson R, Boisvert JL, Benes CH, Zou L (2015). Distinct but concerted roles of ATR, DNA-PK, and Chk1 in countering replication stress during S phase. Mol Cell.

[CR48] Caridi CP, D’Agostino C, Ryu T, Zapotoczny G, Delabaere L, Li X, Khodaverdian VY, Amaral N, Lin E, Rau AR (2018). Nuclear F-actin and myosins drive relocalization of heterochromatic breaks. Nature.

[CR49] Caridi CP, Plessner M, Grosse R, Chiolo I (2019). Nuclear actin filaments in DNA repair dynamics. Nat Cell Biol.

[CR50] Case LB, Zhang X, Ditlev JA, Rosen MK (2019). Stoichiometry controls activity of phase-separated clusters of actin signaling proteins. Science.

[CR51] Cesare AJ, Griffith JD (2004). Telomeric DNA in ALT cells is characterized by free telomeric circles and heterogeneous t-loops. Mol Cell Biol.

[CR52] Chakrabarti R, Ji WK, Stan RV, de Juan Sanz J, Ryan TA, Higgs HN (2018). INF2-mediated actin polymerization at the ER stimulates mitochondrial calcium uptake, inner membrane constriction, and division. J Cell Biol.

[CR53] Cho NW, Dilley RL, Lampson MA, Greenberg RA (2014). Interchromosomal homology searches drive directional ALT telomere movement and synapsis. Cell.

[CR54] Cho S, Vashisth M, Abbas A, Majkut S, Vogel K, Xia Y, Ivanovska IL, Irianto J, Tewari M, Zhu K (2019). Mechanosensing by the lamina protects against nuclear rupture, DNA damage, and Cell-Cycle Arrest. Dev Cell.

[CR55] Churikov D, Charifi F, Eckert-Boulet N, Silva S, Simon MN, Lisby M, Geli V (2016). SUMO-dependent relocalization of eroded telomeres to nuclear pore complexes controls telomere recombination. Cell Rep.

[CR56] Ciccia A, Nimonkar AV, Hu Y, Hajdu I, Achar YJ, Izhar L, Petit SA, Adamson B, Yoon JC, Kowalczykowski SC (2012). Polyubiquitinated PCNA recruits the ZRANB3 translocase to maintain genomic integrity after replication stress. Mol Cell.

[CR57] Clynes D, Jelinska C, Xella B, Ayyub H, Scott C, Mitson M, Taylor S, Higgs DR, Gibbons RJ (2015). Suppression of the alternative lengthening of telomere pathway by the chromatin remodelling factor ATRX. Nat Commun.

[CR58] Cobb AM, De Silva SA, Hayward R, Sek K, Ulferts S, Grosse R, Shanahan CM (2022). Filamentous nuclear actin regulation of PML NBs during the DNA damage response is deregulated by prelamin A. Cell Death Dis.

[CR59] Cook AW, Toseland CP (2021). The roles of nuclear myosin in the DNA damage response. J Biochem.

[CR60] Courtot L, Hoffmann JS, Bergoglio V (2018). The protective role of dormant origins in response to replicative stress. Int J Mol Sci.

[CR61] Cox KE, Maréchal A, Flynn RL (2016). SMARCAL1 resolves replication stress at ALT telomeres. Cell Rep.

[CR62] Crisp M, Liu Q, Roux K, Rattner JB, Shanahan C, Burke B, Stahl PD, Hodzic D (2005). Coupling of the nucleus and cytoplasm: role of the LINC complex. J Cell Biol.

[CR63] Cybulla E, Vindigni A (2023). Leveraging the replication stress response to optimize cancer therapy. Nat Rev Cancer.

[CR64] da Costa A, Chowdhury D, Shapiro GI, D'Andrea AD, Konstantinopoulos PA (2023). Targeting replication stress in cancer therapy. Nat Rev Drug Discov.

[CR65] Dall'Agnese G, Dall'Agnese A, Banani SF, Codrich M, Malfatti MC, Antoniali G, Tell G (2023). Role of condensates in modulating DNA repair pathways and its implication for chemoresistance. J Biol Chem.

[CR66] Datta A, Biswas K, Sommers JA, Thompson H, Awate S, Nicolae CM, Thakar T, Moldovan G-L, Shoemaker RH, Sharan SK (2021). WRN helicase safeguards deprotected replication forks in BRCA2-mutated cancer cells. Nat Commun.

[CR67] de Lange T (2005). Shelterin: the protein complex that shapes and safeguards human telomeres. Genes Dev.

[CR68] Debatisse M, Rosselli F (2019). A journey with common fragile sites: from S phase to telophase. Genes Chromosom Cancer.

[CR69] Deegan TD, Diffley JFX (2016). MCM: one ring to rule them all. Curr Opin Struct Biol.

[CR70] Denais CM, Gilbert RM, Isermann P, McGregor AL, te Lindert M, Weigelin B, Davidson PM, Friedl P, Wolf K, Lammerding J (2016). Nuclear envelope rupture and repair during cancer cell migration. Science.

[CR71] Deng L, Wu RA, Sonneville R, Kochenova OV, Labib K, Pellman D, Walter JC (2019). Mitotic CDK promotes replisome disassembly, fork breakage, and complex DNA rearrangements. Mol Cell.

[CR72] Dewar JM, Walter JC (2017). Mechanisms of DNA replication termination. Nat Rev Mol Cell Biol.

[CR73] Dilley RL, Greenberg RA (2015). ALTernative telomere maintenance and cancer. Trends Cancer.

[CR74] Dilley RL, Verma P, Cho NW, Winters HD, Wondisford AR, Greenberg RA (2016). Break-induced telomere synthesis underlies alternative telomere maintenance. Nature.

[CR75] Diplas BH, He X, Brosnan-Cashman JA, Liu H, Chen LH, Wang Z, Moure CJ, Killela PJ, Loriaux DB, Lipp ES (2018). The genomic landscape of TERT promoter wildtype-IDH wildtype glioblastoma. Nat Commun.

[CR76] Dobbelstein M, Sorensen CS (2015). Exploiting replicative stress to treat cancer. Nat Rev Drug Discov.

[CR77] Dominguez R, Holmes KC (2011). Actin structure and function. Annu Rev Biophys.

[CR78] Donker L, Houtekamer R, Vliem M, Sipieter F, Canever H, Gomez-Gonzalez M, Bosch-Padros M, Pannekoek WJ, Trepat X, Borghi N (2022). A mechanical G2 checkpoint controls epithelial cell division through E-cadherin-mediated regulation of Wee1-Cdk1. Cell Rep.

[CR79] dos Santos A, Cook AW, Gough RE, Schilling M, Olszok NA, Brown I, Wang L, Aaron J, Martin-Fernandez ML, Rehfeldt F (2021). DNA damage alters nuclear mechanics through chromatin reorganization. Nucleic Acids Res.

[CR80] dos Santos Á, Toseland CP (2021). Regulation of nuclear mechanics and the impact on DNA damage. Int J Mol Sci.

[CR81] Douglas ME, Ali FA, Costa A, Diffley JFX (2018). The mechanism of eukaryotic CMG helicase activation. Nature.

[CR82] Draskovic I, Arnoult N, Steiner V, Bacchetti S, Lomonte P, Londoño-Vallejo A (2009). Probing PML body function in ALT cells reveals spatiotemporal requirements for telomere recombination. Proc Natl Acad Sci U S A.

[CR83] D'Souza Y, Lauzon C, Chu TW, Autexier C (2013). Regulation of telomere length and homeostasis by telomerase enzyme processivity. J Cell Sci.

[CR84] Dupont S, Wickström SA (2022). Mechanical regulation of chromatin and transcription. Nat Rev Genet.

[CR85] Durkin SG, Glover TW (2007). Chromosome fragile sites. Annu Rev Genet.

[CR86] El Hage A, French SL, Beyer AL, Tollervey D (2010). Loss of topoisomerase I leads to R-loop-mediated transcriptional blocks during ribosomal RNA synthesis. Genes Dev.

[CR87] Elledge SJ (1996). Cell cycle checkpoints: preventing an identity crisis. Science.

[CR88] Emerson DJ, Zhao PA, Cook AL, Barnett RJ, Klein KN, Saulebekova D, Ge C, Zhou L, Simandi Z, Minsk MK (2022). Cohesin-mediated loop anchors confine the locations of human replication origins. Nature.

[CR89] Epum EA, Haber JE (2022). DNA replication: the recombination connection. Trends Cell Biol.

[CR90] Evrin C, Clarke P, Zech J, Lurz R, Sun J, Uhle S, Li H, Stillman B, Speck C (2009). A double-hexameric MCM2-7 complex is loaded onto origin DNA during licensing of eukaryotic DNA replication. Proc Natl Acad Sci U S A.

[CR91] Fan Y, Köberlin MS, Ratnayeke N, Liu C, Deshpande M, Gerhardt J, Meyer T (2021). LRR1-mediated replisome disassembly promotes DNA replication by recycling replisome components. J Cell Biol.

[CR92] Feng X, Du W, Ding M, Zhao W, Xirefu X, Ma M, Zhuang Y, Fu X, Shen J, Zhang J (2022). Myosin 1D and the branched actin network control the condensation of p62 bodies. Cell Res.

[CR93] Feretzaki M, Pospisilova M, Valador Fernandes R, Lunardi T, Krejci L, Lingner J (2020). RAD51-dependent recruitment of TERRA lncRNA to telomeres through R-loops. Nature.

[CR94] Fernandes RV, Feretzaki M, Lingner J (2021). The makings of TERRA R-loops at chromosome ends. Cell Cycle.

[CR95] Flynn RL, Cox KE, Jeitany M, Wakimoto H, Bryll AR, Ganem NJ, Bersani F, Pineda JR, Suvà ML, Benes CH (2015). Alternative lengthening of telomeres renders cancer cells hypersensitive to ATR inhibitors. Science.

[CR96] Follonier C, Oehler J, Herrador R, Lopes M (2013). Friedreich’s ataxia-associated GAA repeats induce replication-fork reversal and unusual molecular junctions. Nat Struct Mol Biol.

[CR97] Fragkos M, Ganier O, Coulombe P, Méchali M (2015). DNA replication origin activation in space and time. Nat Rev Mol Cell Biol.

[CR98] Frattini C, Promonet A, Alghoul E, Vidal-Eychenie S, Lamarque M, Blanchard MP, Urbach S, Basbous J, Constantinou A (2021). TopBP1 assembles nuclear condensates to switch on ATR signaling. Mol Cell.

[CR99] Gadaleta MC, Noguchi E (2017). Regulation of DNA replication through natural impediments in the eukaryotic genome. Genes.

[CR100] Gaillard H, García-Muse T, Aguilera A (2015). Replication stress and cancer. Nat Rev Cancer.

[CR101] Ganier O, Prorok P, Akerman I, Mechali M (2019). Metazoan DNA replication origins. Curr Opin Cell Biol.

[CR102] García-Gómez S, Reyes A, Martínez-Jiménez MI, Chocrón ES, Mourón S, Terrados G, Powell C, Salido E, Méndez J, Holt IJ (2013). PrimPol, an archaic primase/polymerase operating in human cells. Mol Cell.

[CR103] Garcia-Muse T, Aguilera A (2016). Transcription-replication conflicts: how they occur and how they are resolved. Nat Rev Mol Cell Biol.

[CR104] Gari K, Décaillet C, Delannoy M, Wu L, Constantinou A (2008). Remodeling of DNA replication structures by the branch point translocase FANCM. Proc Natl Acad Sci U S A.

[CR105] Ge XQ, Blow JJ (2010). Chk1 inhibits replication factory activation but allows dormant origin firing in existing factories. J Cell Biol.

[CR106] Gibb B, Ye LF, Gergoudis SC, Kwon Y, Niu H, Sung P, Greene EC (2014). Concentration-dependent exchange of replication protein A on single-stranded DNA revealed by single-molecule imaging. PLoS One.

[CR107] Giles KA, Lamm N, Taberlay PC, Cesare AJ (2022) Three-dimensional chromatin organisation shapes origin activation and replication fork directionality. bioRxiv https://www.biorxiv.org/content/10.1101/2022.06.24.497492v1

[CR108] Glover TW, Wilson TE, Arlt MF (2017). Fragile sites in cancer: more than meets the eye. Nat Rev Cancer.

[CR109] Goelzer M, Goelzer J, Ferguson ML, Neu CP, Uzer G (2021). Nuclear envelope mechanobiology: linking the nuclear structure and function. Nucleus.

[CR110] González-Acosta D, Blanco-Romero E, Ubieto-Capella P, Mutreja K, Míguez S, Llanos S, García F, Muñoz J, Blanco L, Lopes M (2021). PrimPol-mediated repriming facilitates replication traverse of DNA interstrand crosslinks. EMBO J.

[CR111] Graham K, Chandrasekaran A, Wang L, Ladak A, Lafer EM, Rangamani P, Stachowiak JC (2023). Liquid-like VASP condensates drive actin polymerization and dynamic bundling. Nat Phys.

[CR112] Grobelny JV, Godwin AK, Broccoli D (2000). ALT-associated PML bodies are present in viable cells and are enriched in cells in the G(2)/M phase of the cell cycle. J Cell Sci.

[CR113] Grosse R, Copeland JW, Newsome TP, Way M, Treisman R (2003). A role for VASP in RhoA-Diaphanous signalling to actin dynamics and SRF activity. EMBO J.

[CR114] Guilliam TA, Brissett NC, Ehlinger A, Keen BA, Kolesar P, Taylor EM, Bailey LJ, Lindsay HD, Chazin WJ, Doherty AJ (2017). Molecular basis for PrimPol recruitment to replication forks by RPA. Nat Commun.

[CR115] Guilliam TA, Doherty AJ (2017). PrimPol-prime time to reprime. Genes.

[CR116] Gunning PW, Ghoshdastider U, Whitaker S, Popp D, Robinson RC (2015). The evolution of compositionally and functionally distinct actin filaments. J Cell Sci.

[CR117] Haahr P, Hoffmann S, Tollenaere MA, Ho T, Toledo LI, Mann M, Bekker-Jensen S, Raschle M, Mailand N (2016). Activation of the ATR kinase by the RPA-binding protein ETAA1. Nat Cell Biol.

[CR118] Halder S, Ranjha L, Taglialatela A, Ciccia A, Cejka P (2022). Strand annealing and motor driven activities of SMARCAL1 and ZRANB3 are stimulated by RAD51 and the paralog complex. Nucleic Acids Res.

[CR119] Han SS, Wen KK, Garcia-Rubio ML, Wold MS, Aguilera A, Niedzwiedz W, Vyas YM (2022). WASp modulates RPA function on single-stranded DNA in response to replication stress and DNA damage. Nat Commun.

[CR120] Hansen JC, Maeshima K, Hendzel MJ (2021). The solid and liquid states of chromatin. Epigenetics Chromatin.

[CR121] Harding SM, Boiarsky JA, Greenberg RA (2015). ATM dependent silencing links nucleolar chromatin reorganization to DNA damage recognition. Cell Rep.

[CR122] Hari-Gupta Y, Fili N, dos Santos A, Cook AW, Gough RE, Reed HCW, Wang L, Aaron J, Venit T, Wait E (2022). Myosin VI regulates the spatial organisation of mammalian transcription initiation. Nat Commun.

[CR123] Hartwell LH, Weinert TA (1989). Checkpoints: controls that ensure the order of cell cycle events. Science.

[CR124] Heaphy CM, de Wilde RF, Jiao Y, Klein AP, Edil BH, Shi C, Bettegowda C, Rodriguez FJ, Eberhart CG, Hebbar S (2011). Altered telomeres in tumors with ATRX and DAXX mutations. Science.

[CR125] Heaphy CM, Subhawong AP, Hong SM, Goggins MG, Montgomery EA, Gabrielson E, Netto GJ, Epstein JI, Lotan TL, Westra WH (2011). Prevalence of the alternative lengthening of telomeres telomere maintenance mechanism in human cancer subtypes. Am J Pathol.

[CR126] Hobson CM, Kern M, O’Brien ET, Stephens AD, Falvo MR, Superfine R (2020). Correlating nuclear morphology and external force with combined atomic force microscopy and light sheet imaging separates roles of chromatin and lamin A/C in nuclear mechanics. Mol Biol Cell.

[CR127] Hurst V, Shimada K, Gasser SM (2019). Nuclear actin and actin-binding proteins in DNA repair. Trends Cell Biol.

[CR128] Ilves I, Petojevic T, Pesavento JJ, Botchan MR (2010). Activation of the MCM2-7 helicase by association with Cdc45 and GINS proteins. Mol Cell.

[CR129] Irianto J, Pfeifer CR, Bennett RR, Xia Y, Ivanovska IL, Liu AJ, Greenberg RA, Discher DE (2016). Nuclear constriction segregates mobile nuclear proteins away from chromatin. Mol Biol Cell.

[CR130] Irianto J, Xia Y, Pfeifer CR, Athirasala A, Ji J, Alvey C, Tewari M, Bennett RR, Harding SM, Liu AJ (2017). DNA damage follows repair factor depletion and portends genome variation in cancer cells after pore migration. Curr Biol.

[CR131] Isermann P, Lammerding J (2017). Consequences of a tight squeeze: nuclear envelope rupture and repair. Nucleus.

[CR132] Jack A, Kim Y, Strom AR, Lee DSW, Williams B, Schaub JM, Kellogg EH, Finkelstein IJ, Ferro LS, Yildiz A (2022). Compartmentalization of telomeres through DNA-scaffolded phase separation. Dev Cell.

[CR133] Kaminski N, Wondisford AR, Kwon Y, Lynskey ML, Bhargava R, Barroso-González J, García-Expósito L, He B, Xu M, Mellacheruvu D (2022). RAD51AP1 regulates ALT-HDR through chromatin-directed homeostasis of TERRA. Mol Cell.

[CR134] Kannan K, Inagaki A, Silber J, Gorovets D, Zhang J, Kastenhuber ER, Heguy A, Petrini JH, Chan TA, Huse JT (2012). Whole exome sequencing identifies ATRX mutation as a key molecular determinant in lower-grade glioma. Oncotarget.

[CR135] Kastan MB, Bartek J (2004). Cell-cycle checkpoints and cancer. Nature.

[CR136] Kaushal S, Wollmuth CE, Das K, Hile SE, Regan SB, Barnes RP, Haouzi A, Lee SM, House NC, Guyumdzhyan M (2019). Sequence and nuclease requirements for breakage and healing of a structure-forming (AT) n sequence within fragile site FRA16D. Cell Rep.

[CR137] Kemp MG, Akan Z, Yilmaz S, Grillo M, Smith-Roe SL, Kang TH, Cordeiro-Stone M, Kaufmann WK, Abraham RT, Sancar A (2010). Tipin-replication protein A interaction mediates Chk1 phosphorylation by ATR in response to genotoxic stress. J Biol Chem.

[CR138] Kile AC, Chavez DA, Bacal J, Eldirany S, Korzhnev DM, Bezsonova I, Eichman BF, Cimprich KA (2015). HLTF’s ancient HIRAN domain binds 3′ DNA ends to drive replication fork reversal. Mol Cell.

[CR139] Kilic S, Lezaja A, Gatti M, Bianco E, Michelena J, Imhof R, Altmeyer M (2019). Phase separation of 53BP1 determines liquid-like behavior of DNA repair compartments. EMBO J.

[CR140] Kockler ZW, Comeron JM, Malkova A (2021). A unified alternative telomere-lengthening pathway in yeast survivor cells. Mol Cell.

[CR141] Koliada AK, Krasnenkov DS, Vaiserman AM (2015). Telomeric aging: mitotic clock or stress indicator?. Front Genet.

[CR142] Kolinjivadi AM, Sannino V, De Antoni A, Zadorozhny K, Kilkenny M, Técher H, Baldi G, Shen R, Ciccia A, Pellegrini L (2017). Smarcal1-mediated fork reversal triggers Mre11-dependent degradation of nascent DNA in the absence of Brca2 and stable Rad51 nucleofilaments. Mol Cell.

[CR143] Kramara J, Osia B, Malkova A (2018). Break-induced replication: the where, the why, and the how. Trends Genet.

[CR144] Kratz K, de Lange T (2018). Protection of telomeres 1 proteins POT1a and POT1b can repress ATR signaling by RPA exclusion, but binding to CST limits ATR repression by POT1b. J Biol Chem.

[CR145] Krause M, Te Riet J, Wolf K (2013). Probing the compressibility of tumor cell nuclei by combined atomic force–confocal microscopy. Phys Biol.

[CR146] Krippner S, Winkelmeier J, Knerr J, Brandt DT, Virant D, Schwan C, Endesfelder U, Grosse R (2020). Postmitotic expansion of cell nuclei requires nuclear actin filament bundling by α-actinin 4. EMBO Rep.

[CR147] Krishnamoorthy A, Jackson J, Mohamed T, Adolph M, Vindigni A, Cortez D (2021). RADX prevents genome instability by confining replication fork reversal to stalled forks. Mol Cell.

[CR148] Kumagai A, Lee J, Yoo HY, Dunphy WG (2006). TopBP1 activates the ATR-ATRIP complex. Cell.

[CR149] Kumagai A, Shevchenko A, Shevchenko A, Dunphy WG (2010). Treslin collaborates with TopBP1 in triggering the initiation of DNA replication. Cell.

[CR150] Kumagai A, Shevchenko A, Shevchenko A, Dunphy WG (2011). Direct regulation of Treslin by cyclin-dependent kinase is essential for the onset of DNA replication. J Cell Biol.

[CR151] Kumeta M, Yoshimura SH, Harata M, Takeyasu K (2010). Molecular mechanisms underlying nucleocytoplasmic shuttling of actinin-4. J Cell Sci.

[CR152] Laflamme G, Mekhail K (2020). Biomolecular condensates as arbiters of biochemical reactions inside the nucleus. Commun Biol.

[CR153] Lamm N, Read MN, Nobis M, Van Ly D, Page SG, Masamsetti VP, Timpson P, Biro M, Cesare AJ (2020). Nuclear F-actin counteracts nuclear deformation and promotes fork repair during replication stress. Nat Cell Biol.

[CR154] Lamm N, Rogers S, Cesare AJ (2021). Chromatin mobility and relocation in DNA repair. Trends Cell Biol.

[CR155] Law MJ, Lower KM, Voon HP, Hughes JR, Garrick D, Viprakasit V, Mitson M, De Gobbi M, Marra M, Morris A (2010). ATR-X syndrome protein targets tandem repeats and influences allele-specific expression in a size-dependent manner. Cell.

[CR156] Lawrence KS, Tapley EC, Cruz VE, Li Q, Aung K, Hart KC, Schwartz TU, Starr DA, Engebrecht J (2016). LINC complexes promote homologous recombination in part through inhibition of nonhomologous end joining. J Cell Biol.

[CR157] Le S, Moore JK, Haber JE, Greider CW (1999). RAD50 and RAD51 define two pathways that collaborate to maintain telomeres in the absence of telomerase. Genet.

[CR158] Lee C, Hong B, Choi JM, Kim Y, Watanabe S, Ishimi Y, Enomoto T, Tada S, Kim Y, Cho Y (2004). Structural basis for inhibition of the replication licensing factor Cdt1 by geminin. Nature.

[CR159] Lee DSW, Strom AR, Brangwynne CP (2022). The mechanobiology of nuclear phase separation. APL Bioeng.

[CR160] Lee YC, Zhou Q, Chen JJ, Yuan JS (2016). RPA-binding protein ETAA1 is an ATR activator involved in DNA replication stress response. Curr Biol.

[CR161] Lemacon D, Jackson J, Quinet A, Brickner JR, Li S, Yazinski S, You Z, Ira G, Zou L, Mosammaparast N (2017). MRE11 and EXO1 nucleases degrade reversed forks and elicit MUS81-dependent fork rescue in BRCA2-deficient cells. Nat Commun.

[CR162] Leno GH (1992). Regulation of DNA replication by the nuclear envelope. Paper presented at: seminars in CELL BIOLOGY.

[CR163] Leonard AC, Mechali M (2013). DNA replication origins. Cold Spring Harb Perspect Biol.

[CR164] Lewis JS, Gross MH, Sousa J, Henrikus SS, Greiwe JF, Nans A, Diffley JFX, Costa A (2022). Mechanism of replication origin melting nucleated by CMG helicase assembly. Nature.

[CR165] Lezaja A, Altmeyer M (2021). Dealing with DNA lesions: when one cell cycle is not enough. Curr Opin Cell Biol.

[CR166] Lezaja A, Panagopoulos A, Wen Y, Carvalho E, Imhof R, Altmeyer M (2021). RPA shields inherited DNA lesions for post-mitotic DNA synthesis. Nat Commun.

[CR167] Li X, Zhao Q, Liao R, Sun P, Wu X (2003). The SCF(Skp2) ubiquitin ligase complex interacts with the human replication licensing factor Cdt1 and regulates Cdt1 degradation. J Biol Chem.

[CR168] Liao YC, Fernandopulle MS, Wang G, Choi H, Hao L, Drerup CM, Patel R, Qamar S, Nixon-Abell J, Shen Y (2019). RNA granules hitchhike on lysosomes for long-distance transport, Using Annexin A11 as a Molecular Tether. Cell.

[CR169] Limas JC, Cook JG (2019). Preparation for DNA replication: the key to a successful S phase. FEBS Lett.

[CR170] Londoño-Vallejo JA, Der-Sarkissian H, Cazes L, Bacchetti S, Reddel RR (2004). Alternative lengthening of telomeres is characterized by high rates of telomeric exchange. Cancer Res.

[CR171] Lottersberger F, Karssemeijer RA, Dimitrova N, de Lange T (2015). 53BP1 and the LINC Complex promote microtubule-dependent DSB mobility and DNA repair. Cell.

[CR172] Lu R, O'Rourke JJ, Sobinoff AP, Allen JAM, Nelson CB, Tomlinson CG, Lee M, Reddel RR, Deans AJ, Pickett HA (2019). The FANCM-BLM-TOP3A-RMI complex suppresses alternative lengthening of telomeres (ALT). Nat Commun.

[CR173] Lu R, Pickett HA (2022). Telomeric replication stress: the beginning and the end for alternative lengthening of telomeres cancers. Open Biol.

[CR174] Ma CJ, Gibb B, Kwon Y, Sung P, Greene EC (2016). Protein dynamics of human RPA and RAD51 on ssDNA during assembly and disassembly of the RAD51 filament. Nucleic Acids Res.

[CR175] MacDougall CA, Byun TS, Van C, Yee MC, Cimprich KA (2007). The structural determinants of checkpoint activation. Genes Dev.

[CR176] Macheret M, Halazonetis TD (2015). DNA replication stress as a hallmark of cancer. Annu Rev Pathol: Mech Dis.

[CR177] Machida YJ, Hamlin JL, Dutta A (2005). Right place, right time, and only once: replication initiation in metazoans. Cell.

[CR178] Mammoto A, Mammoto T, Ingber DE (2012). Mechanosensitive mechanisms in transcriptional regulation. J Cell Sci.

[CR179] Mangiarotti A, Chen N, Zhao Z, Lipowsky R, Dimova R (2022) Membrane wetting, molding and reticulation by protein condensates. bioRxiv https://www.biorxiv.org/content/10.1101/2022.06.03.494704v3

[CR180] Manthei KA, Keck JL (2013). The BLM dissolvasome in DNA replication and repair. Cell Mol Life Sci.

[CR181] Marchal C, Sima J, Gilbert DM (2019). Control of DNA replication timing in the 3D genome. Nat Rev Mol Cell Biol.

[CR182] Marians KJ (2018). Lesion bypass and the reactivation of stalled replication forks. Annu Rev Biochem.

[CR183] Marnef A, Finoux AL, Arnould C, Guillou E, Daburon V, Rocher V, Mangeat T, Mangeot PE, Ricci EP, Legube G (2019). A cohesin/HUSH- and LINC-dependent pathway controls ribosomal DNA double-strand break repair. Genes Dev.

[CR184] Masai H, Matsumoto S, You Z, Yoshizawa-Sugata N, Oda M (2010). Eukaryotic chromosome DNA replication: where, when, and how?. Annu Rev Biochem.

[CR185] Matthews HK, Bertoli C, de Bruin RAM (2022). Cell cycle control in cancer. Nat Rev Mol Cell Biol.

[CR186] Mazouzi A, Velimezi G, Loizou JI (2014). DNA replication stress: causes, resolution and disease. Exp Cell Res.

[CR187] McCall PM, Srivastava S, Perry SL, Kovar DR, Gardel ML, Tirrell MV (2018). Partitioning and enhanced self-assembly of actin in polypeptide coacervates. Biophys J.

[CR188] McEachern MJ, Haber JE (2006). Break-induced replication and recombinational telomere elongation in yeast. Annu Rev Biochem.

[CR189] Méndez J, Zou-Yang XH, Kim SY, Hidaka M, Tansey WP, Stillman B (2002). Human origin recognition complex large subunit is degraded by ubiquitin-mediated proteolysis after initiation of DNA replication. Mol Cell.

[CR190] Merigliano C, Chiolo I (2021). Multi-scale dynamics of heterochromatin repair. Curr Opin Genet Dev.

[CR191] Mijic S, Zellweger R, Chappidi N, Berti M, Jacobs K, Mutreja K, Ursich S, Ray Chaudhuri A, Nussenzweig A, Janscak P (2017). Replication fork reversal triggers fork degradation in BRCA2-defective cells. Nat Commun.

[CR192] Miller TCR, Locke J, Greiwe JF, Diffley JFX, Costa A (2019). Mechanism of head-to-head MCM double-hexamer formation revealed by cryo-EM. Nature.

[CR193] Min J, Wright WE, Shay JW (2019). Clustered telomeres in phase-separated nuclear condensates engage mitotic DNA synthesis through BLM and RAD52. Genes Dev.

[CR194] Minasi S, Baldi C, Gianno F, Antonelli M, Buccoliero AM, Pietsch T, Massimino M, Buttarelli FR (2021). Alternative lengthening of telomeres in molecular subgroups of paediatric high-grade glioma. Childs Nerv Syst.

[CR195] Mine-Hattab J, Liu SY, Taddei A (2022). Repair foci as liquid phase separation: evidence and limitations. Genes.

[CR196] Minozzo F, Rassier DE, Roberts GCK (2013). Myosin family classification. Encyclopedia of biophysics.

[CR197] Mitrentsi I, Lou J, Kerjouan A, Verigos J, Reina-San-Martin B, Hinde E, Soutoglou E (2022). Heterochromatic repeat clustering imposes a physical barrier on homologous recombination to prevent chromosomal translocations. Mol Cell.

[CR198] Mitrentsi I, Yilmaz D, Soutoglou E (2020). How to maintain the genome in nuclear space. Curr Opin Cell Biol.

[CR199] Moldovan GL, Pfander B, Jentsch S (2007). PCNA, the maestro of the replication fork. Cell.

[CR200] Mourón S, Rodriguez-Acebes S, Martínez-Jiménez MI, García-Gómez S, Chocrón S, Blanco L, Méndez J (2013). Repriming of DNA synthesis at stalled replication forks by human PrimPol. Nat Struct Mol Biol.

[CR201] Nabetani A, Yokoyama O, Ishikawa F (2004). Localization of hRad9, hHus1, hRad1, and hRad17 and caffeine-sensitive DNA replication at the alternative lengthening of telomeres-associated promyelocytic leukemia body. J Biol Chem.

[CR202] Nava MM, Miroshnikova YA, Biggs LC, Whitefield DB, Metge F, Boucas J, Vihinen H, Jokitalo E, Li X, García Arcos JM (2020). Heterochromatin-driven nuclear softening protects the genome against mechanical stress-induced damage. Cell.

[CR203] Neelsen KJ, Lopes M (2015). Replication fork reversal in eukaryotes: from dead end to dynamic response. Nat Rev Mol Cell Biol.

[CR204] Neelsen KJ, Zanini IM, Mijic S, Herrador R, Zellweger R, Ray Chaudhuri A, Creavin KD, Blow JJ, Lopes M (2013). Deregulated origin licensing leads to chromosomal breaks by rereplication of a gapped DNA template. Genes Dev.

[CR205] Nicodemi M, Pombo A (2014). Models of chromosome structure. Curr Opin Cell Biol.

[CR206] Niehrs C, Luke B (2020). Regulatory R-loops as facilitators of gene expression and genome stability. Nat Rev Mol Cell Biol.

[CR207] Nieminuszczy J, Martin PR, Broderick R, Krwawicz J, Kanellou A, Mocanu C, Bousgouni V, Smith C, Wen KK, Woodward BL, Bakal C, Shackley F, Aguilera A, Stewart GS, Vyas YM, Niedzwiedz W (2023) Actin nucleators safeguard replication forks by limiting nascent strand degradation. Nucleic Acids Res 51(12):6337–6354 10.1093/nar/gkad369 PMC10325910 37224534

[CR208] Nimonkar AV, Genschel J, Kinoshita E, Polaczek P, Campbell JL, Wyman C, Modrich P, Kowalczykowski SC (2011). BLM-DNA2-RPA-MRN and EXO1-BLM-RPA-MRN constitute two DNA end resection machineries for human DNA break repair. Genes Dev.

[CR209] Nishitani H, Lygerou Z (2002). Control of DNA replication licensing in a cell cycle. Genes Cells.

[CR210] Nishitani H, Sugimoto N, Roukos V, Nakanishi Y, Saijo M, Obuse C, Tsurimoto T, Nakayama KI, Nakayama K, Fujita M (2006). Two E3 ubiquitin ligases, SCF-Skp2 and DDB1-Cul4, target human Cdt1 for proteolysis. EMBO J.

[CR211] Novak B, Tyson JJ, Gyorffy B, Csikasz-Nagy A (2007). Irreversible cell-cycle transitions are due to systems-level feedback. Nat Cell Biol.

[CR212] Nyberg KA, Michelson RJ, Putnam CW, Weinert TA (2002). Toward maintaining the genome: DNA damage and replication checkpoints. Annu Rev Genet.

[CR213] O’Rourke JJ, Bythell-Douglas R, Dunn EA, Deans AJ (2019). ALT control, delete: FANCM as an anti-cancer target in alternative lengthening of telomeres. Nucleus.

[CR214] O'Donnell M, Langston L, Stillman B (2013). Principles and concepts of DNA replication in bacteria, archaea, and eukarya. Cold Spring Harb Perspect Biol.

[CR215] Okazaki R, Okazaki T, Sakabe K, Sugimoto K, Sugino A (1968). Mechanism of DNA chain growth. I. Possible discontinuity and unusual secondary structure of newly synthesized chains. Proc Natl Acad Sci U S A.

[CR216] Oshidari R, Huang R, Medghalchi M, Tse EYW, Ashgriz N, Lee HO, Wyatt H, Mekhail K (2020). DNA repair by Rad52 liquid droplets. Nat Commun.

[CR217] O'Sullivan RJ, Arnoult N, Lackner DH, Oganesian L, Haggblom C, Corpet A, Almouzni G, Karlseder J (2014). Rapid induction of alternative lengthening of telomeres by depletion of the histone chaperone ASF1. Nat Struct Mol Biol.

[CR218] Özer Ö, Bhowmick R, Liu Y, Hickson ID (2018). Human cancer cells utilize mitotic DNA synthesis to resist replication stress at telomeres regardless of their telomere maintenance mechanism. Oncotarget.

[CR219] Özer Ö, Hickson ID (2018). Pathways for maintenance of telomeres and common fragile sites during DNA replication stress. Open Biol.

[CR220] Pan X, Chen Y, Biju B, Ahmed N, Kong J, Goldenberg M, Huang J, Mohan N, Klosek S, Parsa K (2019). FANCM suppresses DNA replication stress at ALT telomeres by disrupting TERRA R-loops. Sci Rep.

[CR221] Panagopoulos A, Altmeyer M (2021). The hammer and the dance of cell cycle control. Trends Biochem Sci.

[CR222] Panier S, Maric M, Hewitt G, Mason-Osann E, Gali H, Dai A, Labadorf A, Guervilly J-H, Ruis P, Segura-Bayona S (2019). SLX4IP antagonizes promiscuous BLM activity during ALT maintenance. Mol Cell.

[CR223] Parisis N, Krasinska L, Harker B, Urbach S, Rossignol M, Camasses A, Dewar J, Morin N, Fisher D (2017). Initiation of DNA replication requires actin dynamics and formin activity. EMBO J.

[CR224] Parker MW, Botchan MR, Berger JM (2017). Mechanisms and regulation of DNA replication initiation in eukaryotes. Crit Rev Biochem Mol Biol.

[CR225] Pefani DE, Latusek R, Pires I, Grawenda AM, Yee KS, Hamilton G, van der Weyden L, Esashi F, Hammond EM, O'Neill E (2014). RASSF1A-LATS1 signalling stabilizes replication forks by restricting CDK2-mediated phosphorylation of BRCA2. Nat Cell Biol.

[CR226] Pefani DE, O'Neill E (2016). Hippo pathway and protection of genome stability in response to DNA damage. FEBS J.

[CR227] Pennycook BR, Barr AR (2020). Restriction point regulation at the crossroads between quiescence and cell proliferation. FEBS Lett.

[CR228] Perez-Gonzalez NA, Rochman ND, Yao K, Tao J, Le MT, Flanary S, Sablich L, Toler B, Crentsil E, Takaesu F (2019). YAP and TAZ regulate cell volume. J Cell Biol.

[CR229] Petermann E, Lan L, Zou L (2022). Sources, resolution and physiological relevance of R-loops and RNA-DNA hybrids. Nat Rev Mol Cell Biol.

[CR230] Petropoulos M, Tsaniras SC, Taraviras S, Lygerou Z (2019). Replication licensing aberrations, replication stress, and genomic instability. Trends Biochem Sci.

[CR231] Pfeifer CR, Xia Y, Zhu K, Liu D, Irianto J, García VMM, Millán LMS, Niese B, Harding S, Deviri D (2018). Constricted migration increases DNA damage and independently represses cell cycle. Mol Biol Cell.

[CR232] Pfitzer L, Moser C, Gegenfurtner F, Arner A, Foerster F, Atzberger C, Zisis T, Kubisch-Dohmen R, Busse J, Smith R (2019). Targeting actin inhibits repair of doxorubicin-induced DNA damage: a novel therapeutic approach for combination therapy. Cell Death Dis.

[CR233] Piberger AL, Bowry A, Kelly RDW, Walker AK, González-Acosta D, Bailey LJ, Doherty AJ, Méndez J, Morris JR, Bryant HE (2020). PrimPol-dependent single-stranded gap formation mediates homologous recombination at bulky DNA adducts. Nat Commun.

[CR234] Pinzaru AM, Kareh M, Lamm N, Lazzerini-Denchi E, Cesare AJ, Sfeir A (2020). Replication stress conferred by POT1 dysfunction promotes telomere relocalization to the nuclear pore. Genes Dev.

[CR235] Plessner M, Grosse R (2019). Dynamizing nuclear actin filaments. Curr Opin Cell Biol.

[CR236] Polo SE, Almouzni G (2015). Chromatin dynamics after DNA damage: the legacy of the access–repair–restore model. DNA Repair.

[CR237] Poole LA, Cortez D (2017). Functions of SMARCAL1, ZRANB3, and HLTF in maintaining genome stability. Crit Rev Biochem Mol Biol.

[CR238] Poole LA, Zhao R, Glick GG, Lovejoy CA, Eischen CM, Cortez D (2015). SMARCAL1 maintains telomere integrity during DNA replication. Proc Natl Acad Sci U S A.

[CR239] Priego Moreno S, Jones RM, Poovathumkadavil D, Scaramuzza S, Gambus A (2019). Mitotic replisome disassembly depends on TRAIP ubiquitin ligase activity. Life Science Alliance.

[CR240] Prioleau MN, MacAlpine DM (2016). DNA replication origins-where do we begin?. Genes Dev.

[CR241] Quinet A, Lemaçon D, Vindigni A (2017). Replication fork reversal: players and guardians. Mol Cell.

[CR242] Quinet A, Tirman S, Cybulla E, Meroni A, Vindigni A (2021). To skip or not to skip: choosing repriming to tolerate DNA damage. Mol Cell.

[CR243] Raab M, Gentili M, de Belly H, Thiam H-R, Vargas P, Jimenez AJ, Lautenschlaeger F, Voituriez R, Lennon-Duménil A-M, Manel N (2016). ESCRT III repairs nuclear envelope ruptures during cell migration to limit DNA damage and cell death. Science.

[CR244] Rawal CC, Caridi CP, Chiolo I (2019). Actin' between phase separated domains for heterochromatin repair. DNA Repair (Amst).

[CR245] Remus D, Beuron F, Tolun G, Griffith JD, Morris EP, Diffley JF (2009). Concerted loading of Mcm2-7 double hexamers around DNA during DNA replication origin licensing. Cell.

[CR246] Roake CM, Artandi SE (2020). Regulation of human telomerase in homeostasis and disease. Nat Rev Mol Cell Biol.

[CR247] Root H, Larsen A, Komosa M, Al-Azri F, Li R, Bazett-Jones DP, Stephen Meyn M (2016). FANCD2 limits BLM-dependent telomere instability in the alternative lengthening of telomeres pathway. Hum Mol Genet.

[CR248] Rubin SM, Sage J, Skotheim JM (2020). Integrating old and new paradigms of G1/S control. Mol Cell.

[CR249] Saayman X, Graham E, Nathan WJ, Nussenzweig A, Esashi F (2023). Centromeres as universal hotspots of DNA breakage, driving RAD51-mediated recombination during quiescence. Mol Cell.

[CR250] Saldivar JC, Cortez D, Cimprich KA (2017). The essential kinase ATR: ensuring faithful duplication of a challenging genome. Nat Rev Mol Cell Biol.

[CR251] Salvi JS, Chan JN, Szafranski K, Liu TT, Wu JD, Olsen JB, Khanam N, Poon BP, Emili A, Mekhail K (2014). Roles for Pbp1 and caloric restriction in genome and lifespan maintenance via suppression of RNA-DNA hybrids. Dev Cell.

[CR252] Sarni D, Kerem B (2016). The complex nature of fragile site plasticity and its importance in cancer. Curr Opin Cell Biol.

[CR253] Sarshad A, Sadeghifar F, Louvet E, Mori R, Bohm S, Al-Muzzaini B, Vintermist A, Fomproix N, Ostlund AK, Percipalle P (2013). Nuclear myosin 1c facilitates the chromatin modifications required to activate rRNA gene transcription and cell cycle progression. PLoS Genet.

[CR254] Saxena S, Zou L (2022). Hallmarks of DNA replication stress. Mol Cell.

[CR255] Schrank B, Gautier J (2019). Assembling nuclear domains: lessons from DNA repair. J Cell Biol.

[CR256] Schrank BR, Aparicio T, Li Y, Chang W, Chait BT, Gundersen GG, Gottesman ME, Gautier J (2018). Nuclear ARP2/3 drives DNA break clustering for homology-directed repair. Nature.

[CR257] Schreiber KH, Kennedy BK (2013). When lamins go bad: nuclear structure and disease. Cell.

[CR258] Schwartzentruber J, Korshunov A, Liu XY, Jones DT, Pfaff E, Jacob K, Sturm D, Fontebasso AM, Quang DA, Tönjes M (2012). Driver mutations in histone H3.3 and chromatin remodelling genes in paediatric glioblastoma. Nature.

[CR259] Scully R, Panday A, Elango R, Willis NA (2019). DNA double-strand break repair-pathway choice in somatic mammalian cells. Nat Rev Mol Cell Biol.

[CR260] Shay JW, Wright WE (2019). Telomeres and telomerase: three decades of progress. Nat Rev Genet.

[CR261] Shimamoto Y, Tamura S, Masumoto H, Maeshima K (2017). Nucleosome–nucleosome interactions via histone tails and linker DNA regulate nuclear rigidity. Mol Biol Cell.

[CR262] Shin Y, Berry J, Pannucci N, Haataja MP, Toettcher JE, Brangwynne CP (2017). Spatiotemporal control of intracellular phase transitions using light-activated optodroplets. Cell.

[CR263] Shokrollahi M, Stanic M, Hundal A, Chan JNY, Urman D, Hakem A, Garcia RE, Hao J, Maass PG, Dickson BC et al (2023) DNA double-strand break-capturing nuclear envelope tubules drive DNA repair. bioRxiv https://www.biorxiv.org/content/10.1101/2023.05.07.539750v1 10.1038/s41594-024-01286-7 38632359

[CR264] Shorrocks A-MK, Jones SE, Tsukada K, Morrow CA, Belblidia Z, Shen J, Vendrell I, Fischer R, Kessler BM, Blackford AN (2021). The Bloom syndrome complex senses RPA-coated single-stranded DNA to restart stalled replication forks. Nat Commun.

[CR265] Silva B, Arora R, Bione S, Azzalin CM (2021). TERRA transcription destabilizes telomere integrity to initiate break-induced replication in human ALT cells. Nat Commun.

[CR266] Silva B, Pentz R, Figueira AM, Arora R, Lee YW, Hodson C, Wischnewski H, Deans AJ, Azzalin CM (2019). FANCM limits ALT activity by restricting telomeric replication stress induced by deregulated BLM and R-loops. Nat Commun.

[CR267] Sobinoff AP, Allen JA, Neumann AA, Yang SF, Walsh ME, Henson JD, Reddel RR, Pickett HA (2017). BLM and SLX4 play opposing roles in recombination-dependent replication at human telomeres. EMBO J.

[CR268] Sonneville R, Bhowmick R, Hoffmann S, Mailand N, Hickson ID, Labib K (2019). TRAIP drives replisome disassembly and mitotic DNA repair synthesis at sites of incomplete DNA replication. eLife.

[CR269] Soranno A, Incicco JJ, De Bona P, Tomko EJ, Galburt EA, Holehouse AS, Galletto R (2022). Shelterin components modulate nucleic acids condensation and phase separation in the context of telomeric DNA. J Mol Biol.

[CR270] Spagnol ST, Armiger TJ, Dahl KN (2016). Mechanobiology of chromatin and the nuclear interior. Cell Mol Bioeng.

[CR271] Spegg V, Altmeyer M (2021). Biomolecular condensates at sites of DNA damage: more than just a phase. DNA Repair (Amst).

[CR272] Spegg V, Panagopoulos A, Stout M, Krishnan A, Reginato G, Imhof R, Roschitzki B, Cejka P, Altmeyer M (2023). Phase separation properties of RPA combine high-affinity ssDNA binding with dynamic condensate functions at telomeres. Nat Struct Mol Biol.

[CR273] Spichal M, Brion A, Herbert S, Cournac A, Marbouty M, Zimmer C, Koszul R, Fabre E (2016). Evidence for a dual role of actin in regulating chromosome organization and dynamics in yeast. J Cell Sci.

[CR274] Spichal M, Fabre E (2017). The emerging role of the cytoskeleton in chromosome dynamics. Front Genet.

[CR275] Stavropoulos DJ, Bradshaw PS, Li X, Pasic I, Truong K, Ikura M, Ungrin M, Meyn MS (2002). The Bloom syndrome helicase BLM interacts with TRF2 in ALT cells and promotes telomeric DNA synthesis. Hum Mol Genet.

[CR276] Stephens AD, Banigan EJ, Adam SA, Goldman RD, Marko JF (2017). Chromatin and lamin A determine two different mechanical response regimes of the cell nucleus. Mol Biol Cell.

[CR277] Stephens AD, Banigan EJ, Marko JF (2019). Chromatin’s physical properties shape the nucleus and its functions. Curr Opin Cell Biol.

[CR278] Strickfaden H, Tolsma TO, Sharma A, Underhill DA, Hansen JC, Hendzel MJ (2020). Condensed chromatin behaves like a solid on the mesoscale in vitro and in living cells. Cell.

[CR279] Strom AR, Kim Y, Zhao H, Orlovsky N, Chang Y-C, Košmrlj A, Storm C, Brangwynne CP (2023) Condensate-driven interfacial forces reposition DNA loci and measure chromatin viscoelasticity. bioRxiv https://www.biorxiv.org/content/10.1101/2023.02.27.530281v1 10.1016/j.cell.2024.07.034 39168125

[CR280] Sturzenegger A, Burdova K, Kanagaraj R, Levikova M, Pinto C, Cejka P, Janscak P (2014). DNA2 cooperates with the WRN and BLM RecQ helicases to mediate long-range DNA end resection in human cells. J Biol Chem.

[CR281] Suski JM, Ratnayeke N, Braun M, Zhang T, Strmiska V, Michowski W, Can G, Simoneau A, Snioch K, Cup M (2022). CDC7-independent G1/S transition revealed by targeted protein degradation. Nature.

[CR282] Swartz RK, Rodriguez EC, King MC (2014). A role for nuclear envelope-bridging complexes in homology-directed repair. Mol Biol Cell.

[CR283] Taglialatela A, Alvarez S, Leuzzi G, Sannino V, Ranjha L, Huang JW, Madubata C, Anand R, Levy B, Rabadan R (2017). Restoration of replication fork stability in BRCA1- and BRCA2-deficient cells by inactivation of SNF2-family fork remodelers. Mol Cell.

[CR284] Tarsounas M, Sung P (2020). The antitumorigenic roles of BRCA1-BARD1 in DNA repair and replication. Nat Rev Mol Cell Biol.

[CR285] Taylor MRG, Yeeles JTP (2018). The initial response of a eukaryotic replisome to DNA damage. Mol Cell.

[CR286] Taylor MRG, Yeeles JTP (2019). Dynamics of replication fork progression following helicase-polymerase uncoupling in eukaryotes. J Mol Biol.

[CR287] Thangavel S, Berti M, Levikova M, Pinto C, Gomathinayagam S, Vujanovic M, Zellweger R, Moore H, Lee EH, Hendrickson EA (2015). DNA2 drives processing and restart of reversed replication forks in human cells. J Cell Biol.

[CR288] Tirman S, Cybulla E, Quinet A, Meroni A, Vindigni A (2021). PRIMPOL ready, set, reprime!. Crit Rev Biochem Mol Biol.

[CR289] Tirman S, Quinet A, Wood M, Meroni A, Cybulla E, Jackson J, Pegoraro S, Simoneau A, Zou L, Vindigni A (2021). Temporally distinct post-replicative repair mechanisms fill PRIMPOL-dependent ssDNA gaps in human cells. Mol Cell.

[CR290] Toledo LI, Altmeyer M, Rask MB, Lukas C, Larsen DH, Povlsen LK, Bekker-Jensen S, Mailand N, Bartek J, Lukas J (2013). ATR prohibits replication catastrophe by preventing global exhaustion of RPA. Cell.

[CR291] Torres-Rosell J, Sunjevaric I, De Piccoli G, Sacher M, Eckert-Boulet N, Reid R, Jentsch S, Rothstein R, Aragon L, Lisby M (2007). The Smc5-Smc6 complex and SUMO modification of Rad52 regulates recombinational repair at the ribosomal gene locus. Nat Cell Biol.

[CR292] Tortora MMC, Brennan L, Karpen G, Jost D (2022) Liquid-liquid phase separation recapitulates the thermodynamics and kinetics of heterochromatin formation. bioRxiv https://www.biorxiv.org/content/10.1101/2022.07.11.499635v2 10.1073/pnas.2211855120 PMC10438847 37549295

[CR293] Tsekrekou M, Stratigi K, Chatzinikolaou G (2017). The nucleolus: In Genome maintenance and repair. Int J Mol Sci.

[CR294] Tsouroula K, Furst A, Rogier M, Heyer V, Maglott-Roth A, Ferrand A, Reina-San-Martin B, Soutoglou E (2016). Temporal and spatial uncoupling of DNA double strand break repair pathways within mammalian heterochromatin. Mol Cell.

[CR295] Turkalo TK, Maffia A, Schabort JJ, Regalado SG, Bhakta M, Blanchette M, Spierings DCJ, Lansdorp PM, Hockemeyer D (2023). A non-genetic switch triggers alternative telomere lengthening and cellular immortalization in ATRX deficient cells. Nat Commun.

[CR296] Udugama M, Vinod B, Chan FL, Hii L, Garvie A, Collas P, Kalitsis P, Steer D, Das PP, Tripathi P (2022). Histone H3.3 phosphorylation promotes heterochromatin formation by inhibiting H3K9/K36 histone demethylase. Nucleic Acids Res.

[CR297] Ulferts S, Prajapati B, Grosse R, Vartiainen MK (2021). Emerging properties and functions of actin and actin filaments inside the nucleus. Cold Spring Harb Perspect Biol.

[CR298] Verma P, Dilley RL, Zhang T, Gyparaki MT, Li Y, Greenberg RA (2019). RAD52 and SLX4 act nonepistatically to ensure telomere stability during alternative telomere lengthening. Genes Dev.

[CR299] Villa F, Fujisawa R, Ainsworth J, Nishimura K, Lie ALM, Lacaud G, Labib KP (2021). CUL2(LRR1) , TRAIP and p97 control CMG helicase disassembly in the mammalian cell cycle. EMBO Rep.

[CR300] Vouzas AE, Gilbert DM (2021). Mammalian DNA replication timing. Cold Spring Harb Perspect Biol.

[CR301] Vujanovic M, Krietsch J, Raso MC, Terraneo N, Zellweger R, Schmid JA, Taglialatela A, Huang JW, Holland CL, Zwicky K (2017). Replication fork slowing and reversal upon DNA damage require PCNA polyubiquitination and ZRANB3 DNA translocase activity. Mol Cell.

[CR302] Wang S, Stoops E, Cp U, Markus B, Reuveny A, Ordan E, Volk T (2018). Mechanotransduction via the LINC complex regulates DNA replication in myonuclei. J Cell Biol.

[CR303] Warmerdam DO, Wolthuis RM (2019). Keeping ribosomal DNA intact: a repeating challenge. Chromosom Res.

[CR304] Wei M, Fan X, Ding M, Li R, Shao S, Hou Y, Meng S, Tang F, Li C, Sun Y (2020). Nuclear actin regulates inducible transcription by enhancing RNA polymerase II clustering. Sci Adv.

[CR305] Weston R, Peeters H, Ahel D (2012). ZRANB3 is a structure-specific ATP-dependent endonuclease involved in replication stress response. Genes Dev.

[CR306] Whalen JM, Dhingra N, Wei L, Zhao X, Freudenreich CH (2020). Relocation of collapsed forks to the nuclear pore complex depends on sumoylation of DNA repair proteins and permits Rad51 association. Cell Rep.

[CR307] Wiegand T, Hyman AA (2020). Drops and fibers - how biomolecular condensates and cytoskeletal filaments influence each other. Emerg Top Life Sci.

[CR308] Wohlschlegel JA, Dwyer BT, Dhar SK, Cvetic C, Walter JC, Dutta A (2000). Inhibition of eukaryotic DNA replication by geminin binding to Cdt1. Science.

[CR309] Wong RP, Petriukov K, Ulrich HD (2021). Daughter-strand gaps in DNA replication - substrates of lesion processing and initiators of distress signalling. DNA Repair.

[CR310] Woolner S, Bement WM (2009). Unconventional myosins acting unconventionally. Trends Cell Biol.

[CR311] Wu G, Lee WH, Chen PL (2000). NBS1 and TRF1 colocalize at promyelocytic leukemia bodies during late S/G2 phases in immortalized telomerase-negative cells. Implication of NBS1 in alternative lengthening of telomeres. J Biol Chem.

[CR312] Xia Y, Ivanovska IL, Zhu K, Smith L, Irianto J, Pfeifer CR, Alvey CM, Ji J, Liu D, Cho S (2018). Nuclear rupture at sites of high curvature compromises retention of DNA repair factors. J Cell Biol.

[CR313] Xia Y, Pfeifer CR, Zhu K, Irianto J, Liu D, Pannell K, Chen EJ, Dooling LJ, Tobin MP, Wang M (2019). Rescue of DNA damage after constricted migration reveals a mechano-regulated threshold for cell cycle. J Cell Biol.

[CR314] Xie G, Walker RR, Irianto J (2020). Nuclear mechanosensing: mechanism and consequences of a nuclear rupture. Mutat Res.

[CR315] Yadav T, Zhang J-M, Ouyang J, Leung W, Simoneau A, Zou L (2022). TERRA and RAD51AP1 promote alternative lengthening of telomeres through an R- to D-loop switch. Mol Cell.

[CR316] Yamazaki H, Takagi M, Kosako H, Hirano T, Yoshimura SH (2022). Cell cycle-specific phase separation regulated by protein charge blockiness. Nat Cell Biol.

[CR317] Yan VT, Narayanan A, Wiegand T, Julicher F, Grill SW (2022). A condensate dynamic instability orchestrates actomyosin cortex activation. Nature.

[CR318] Yanagi K-I, Mizuno T, You Z, Hanaoka F (2002). Mouse geminin inhibits not only Cdt1-MCM6 interactions but also a novel intrinsic Cdt1 DNA binding activity. J Biol Chem.

[CR319] Ye G, Yang Q, Lei X, Zhu X, Li F, He J, Chen H, Ling R, Zhang H, Lin T (2020). Nuclear MYH9-induced CTNNB1 transcription, targeted by staurosporin, promotes gastric cancer cell anoikis resistance and metastasis. Theranostics.

[CR320] Yeager TR, Neumann AA, Englezou A, Huschtscha LI, Noble JR, Reddel RR (1999). Telomerase-negative immortalized human cells contain a novel type of promyelocytic leukemia (PML) body. Cancer Res.

[CR321] Yeeles JT, Poli J, Marians KJ, Pasero P (2013). Rescuing stalled or damaged replication forks. Cold Spring Harb Perspect Biol.

[CR322] Yilmaz D, Furst A, Meaburn K, Lezaja A, Wen Y, Altmeyer M, Reina-San-Martin B, Soutoglou E (2021). Activation of homologous recombination in G1 preserves centromeric integrity. Nature.

[CR323] Zeman MK, Cimprich KA (2014). Causes and consequences of replication stress. Nat Cell Biol.

[CR324] Zhang H, Zhao R, Tones J, Liu M, Dilley RL, Chenoweth DM, Greenberg RA, Lampson MA (2020). Nuclear body phase separation drives telomere clustering in ALT cancer cells. Mol Biol Cell.

[CR325] Zhang JM, Genois MM, Ouyang J, Lan L, Zou L (2021). Alternative lengthening of telomeres is a self-perpetuating process in ALT-associated PML bodies. Mol Cell.

[CR326] Zhang JM, Yadav T, Ouyang J, Lan L, Zou L (2019). Alternative lengthening of telomeres through two distinct break-induced replication pathways. Cell Rep.

[CR327] Zhang J-M, Zou L (2020). Alternative lengthening of telomeres: from molecular mechanisms to therapeutic outlooks. Cell Biosci.

[CR328] Zhang Y, Lee DSW, Meir Y, Brangwynne CP, Wingreen NS (2021). Mechanical frustration of phase separation in the cell nucleus by chromatin. Phys Rev Lett.

[CR329] Zhou Y, Kumari D, Sciascia N, Usdin K (2016). CGG-repeat dynamics and FMR1 gene silencing in fragile X syndrome stem cells and stem cell-derived neurons. Molecular Autism.

[CR330] Zhou Y, Meng X, Zhang S, Lee EY, Lee MY (2012). Characterization of human DNA polymerase delta and its subassemblies reconstituted by expression in the MultiBac system. PLoS One.

[CR331] Zhu S, Hou J, Gao H, Hu Q, Kloeber JA, Huang J, Zhao F, Zhou Q, Luo K, Wu Z (2023). SUMOylation of HNRNPA2B1 modulates RPA dynamics during unperturbed replication and genotoxic stress responses. Mol Cell.

[CR332] Zidovska A (2020). Chromatin: Liquid or Solid?. Cell.

[CR333] Zou L, Elledge SJ (2003). Sensing DNA damage through ATRIP recognition of RPA-ssDNA complexes. Science.

[CR334] Zou L, Stillman B (2000). Assembly of a complex containing Cdc45p, replication protein A, and Mcm2p at replication origins controlled by S-phase cyclin-dependent kinases and Cdc7p-Dbf4p kinase. Mol Cell Biol.

